# Time-Dependent Resonant Inelastic X-ray Scattering of Pyrazine at the Nitrogen K-Edge: A Quantum Dynamics Approach

**DOI:** 10.1021/acs.jctc.3c01259

**Published:** 2024-02-05

**Authors:** Antonia Freibert, David Mendive-Tapia, Nils Huse, Oriol Vendrell

**Affiliations:** † Department of Physics, University of Hamburg, Luruper Chaussee 149, 22761 Hamburg, Germany; ‡ Theoretical Chemistry, Institute of Physical Chemistry, Heidelberg University, Im Neuenheimer Feld 229, 69120 Heidelberg, Germany

## Abstract

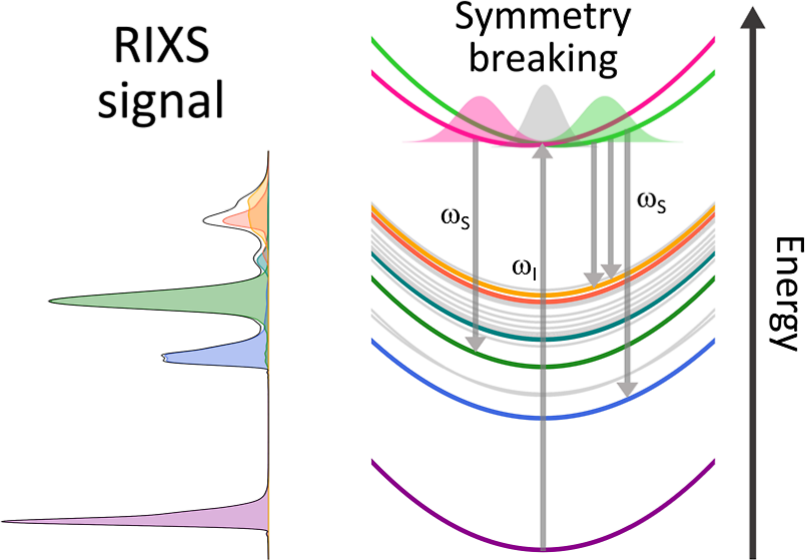

We calculate resonant inelastic X-ray scattering spectra of pyrazine at the nitrogen K-edge in the time domain including wavepacket dynamics in both the valence and core-excited state manifolds. Upon resonant excitation, we observe ultrafast non-adiabatic population transfer between core-excited states within the core-hole lifetime, leading to molecular symmetry distortions. Importantly, our time-domain approach inherently contains the ability to manipulate the dynamics of this process by detuning the excitation energy, which effectively shortens the scattering duration. We also explore the impact of pulsed incident X-ray radiation, which provides a foundation for state-of-the-art time-resolved experiments with coherent pulsed light sources.

## Introduction

The development of high-brilliance synchroton X-ray radiation sources^1−3^ and X-ray free electron lasers^4−7^ has enabled techniques that require high photon flux such as resonant inelastic X-ray scattering (RIXS)^8^ and its extension into the ultrafast time domain.^9−17^ RIXS constitutes a Raman scattering process in which the system is resonantly excited into short-lived core-hole states and spontaneously decays back to the electronic ground and excited states.^8,18^ This technique combines the element specificity of core-level spectroscopy with the ability to reach valence-excited states across a wide spectral range (>20 eV) even in the condensed phase^19^ and at a spectral resolution that is not limited by the core-hole lifetime broadening, making it a versatile and promising tool to study the local electronic structure in complex molecular systems. RIXS to ground and valence-excited states has already been applied in various areas such as the nature of hydrogen-bond interactions,^20−22^ the photochemistry of transition-metal complexes,^23−29^ ultrafast dynamics of molecules on surfaces,^30,31^ proton transfer dynamics,^13,32,33^ or core-excited state dynamics.^34−36^

Within the dipole approximation, RIXS follows electronic symmetry selection rules that offer valuable insights into the symmetry of occupied and unoccupied orbitals in molecules.^37−40^ For instance, by strictly adhering to the dipole selection rules during both the absorption and emission steps, the parity of the system must remain unchanged throughout the RIXS process. However, molecules with equivalent atoms always possess multicenter core orbitals that are delocalized over these atoms, resulting in nearly degenerate core-excited states, commonly referred to as the core-hole localization problem.^41−46^ These states couple vibronically through a non-totally symmetric normal mode, thus corresponding to a symmetry-allowed conical intersection^47^ and resulting in a final localization of the core holes.^18,37,48,49^ This dynamical distortion of symmetry can enable transitions that are otherwise electronically symmetry-forbidden^38,39,50^ and significantly impact the intensity of their RIXS signal.^49^

One effective method to control or even prevent symmetry breaking in RIXS is to adjust the excitation energy to below the X-ray absorption resonance.^51,52^ More generally, this detuning of the excitation energy allows for the manipulation of the effective lifetime in the core-excited states and, consequently, the scattering duration.^51,53,54^ Hence, by varying the detuning, it is possible to control and manipulate dynamical processes within the femtosecond scattering duration, such as dissociation,^53,55^ vibrational collapse,^56,57^ or symmetry distortion.^36,58−60^

There are different theoretical approaches to simulating RIXS spectra, which can be broadly classified into time-independent and time-dependent methods. In general, the quantum mechanical description of X-ray Raman scattering is based on second-order time-dependent perturbation theory which gives rise to the well-known Kramers–Heisenberg–Dirac (KHD) expression for the polarizabilty tensor in the frequency domain.^61−63^ Starting from this sum-over-states form, time-independent methods rely on an eigenstate representation of the system, ideally considering all molecular vibronic states that contribute to the resonance effect.^64,65^ Although various efficient techniques have been developed,^66−69^ as the size and complexity of the molecular system increase, summing over all the eigenstates of the vibrational modes in the molecular excited state becomes rapidly computationally demanding. Therefore, time-dependent strategies based on wave packet propagation on the excited state manifold have been proposed as alternatives, where the scattering amplitude is determined by the half-Fourier transform of the time-dependent overlap between the final and initial vibrational states.^63,70^ Various levels of theory have been employed, including real-time propagation,^71,72^ Green’s function techniques,^73,74^ and methods based on time-dependent density functional theory,^75,76^ demonstrating the extensive potential and applicability of time-domain formalisms.

In the present work, we examine the RIXS process of pyrazine at the nitrogen K-edge by using a comprehensive time-domain approach. We employ a full quantum mechanical treatment of both, the nuclear and electronic degrees of freedom, under symmetry-selective excitations in a diabatic representation of the Hamiltonian within the multiconfiguration time-dependent Hartree (MCTDH)^77^ framework. To accurately depict dynamic processes occurring within the ultrashort core-hole lifetime and their manipulation through changes in the excitation frequency, we explicitly incorporate nuclear motion on the core-excited state manifold. Additionally, we explicitly introduce an arbitrary coherent spectral distribution, e.g., an incoming ultrashort X-ray pulse, and investigate how it manifests in the resulting spectra, thereby enabling an optimal interplay between theory and experiment.

## Methodology

### Model Hamiltonian

The full Hamiltonian **H** employed in the nuclear quantum dynamics simulations consists of a molecular Hamiltonian **H**_mol_ supplemented by the interaction with an external electromagnetic field **H**_int_ acting as a time-dependent perturbation
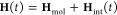
1Following the semi-classical *ansatz* of ref (78), the coupling to the external field is assumed to be accurately captured by the dipole approximation
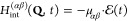
2where  is the electric field of the photon beam and μ_αβ_ is the transition dipole moment between the electronic states |α⟩ and |β⟩, and where the Condon approximation and a single polarization direction are assumed. Within the rotating wave approximation, the electric field is represented by
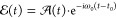
3with the temporal envelope function  of the laser pulse centered at *t*_0_ and with carrier frequency ω_0_. In particular, we refer to a continuous wave (CW) experiment if .

As in ref (78), we decouple the valence and core-excited states,^79,80^ yielding the following matrix representation of the molecular Hamiltonian
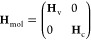
4where **H**_v_ and **H**_c_ are sub-Hamiltonians acting on the manifolds of the valence and core-excited electronic states, respectively. In both subspaces, a vibronic coupling model up to second order^47,48,81^ is employed, leading to coupled potential energy surfaces (PESs) in a diabatic representation.^82,83^ In this framework, each submatrix in eq 4 is expressed as
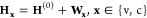
5where the zeroth-order Hamiltonian is constituted by the ground-state Hamiltonian in the harmonic approximation
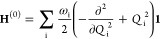
6with ω_i_ representing the frequency of mode *Q*_i_. The diabatic potential matrix **W**_**x**_ covers all changes in the excited state surfaces compared to the ground state, whose matrix elements are in this work given by
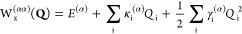
7
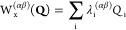
8with the vertical energy displacement *E*^(α)^ of the α-th electronic state, the linear intrastate coupling constant κ_i_^(α)^ related to the gradients of the adiabatic potentials at the Franck–Condon point, the quadratic intrastate coupling constant γ_i_^(α)^ enclosing changes in the frequencies, and the linear interstate coupling constants λ_i_^(αβ)^ belonging to the non-adiabatic interaction between the α-th and β-th electronic states under displacements of the *Q*_i_ mode.

For strongly anharmonic modes, the harmonic expression of the diabatic potentials is replaced by Morse potentials
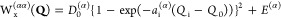
9where *D*_0_^(α)^ denotes the state-specific dissociation energy, *α*_i_^(α)^ defines the curvature of the potential, and *Q*_0_ is the equilibrium position.

The parameters required to describe the diabatic molecular Hamiltonian were obtained from electronic structure calculations performed at the coupled cluster singles and doubles (CCSD) level of theory^84^ and its extensions for excited states using Dunning’s correlation consistent basis set aug-cc-pVDZ.^85^ Ground-state geometry optimization and normal mode evaluation were computed using the quantum chemistry software package Gaussian.^86^ Excited-state calculations were performed using the quantum chemistry software package Q-Chem^87^ where adiabatic energies, energy gradients, and non-adiabatic coupling terms for the valence-excited states were obtained employing the equation-of-motion (EOM-) CCSD approach^88^ while core-excited state properties were computed using the frozen-core/core–valence-separated (fc-CVS-) EOM-CCSD method.^89^ The latter two quantities were projected from Cartesian coordinates to the normal mode coordinate representation; for this purpose, the VCHam tools within the Heidelberg MCTDH package were used^90^
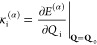
10
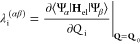
11where **H**_el_ denotes the electronic Hamiltonian. In the case of the most relevant electronic states, the corresponding excited state parameters were refitted through a least-squares procedure applied to a series of ab initio single-point energy calculations along each normal mode.

### Derivation of Spectral Properties

The underlying mechanism of RIXS is a two-photon process that first involves interaction with an incident X-ray beam, creating an evolving wavepacket on the core-excited state manifold, followed by spontaneous emission of a photon. The time-dependent picture of RIXS can hence be retrieved by second-order time-dependent perturbation theory for the light–matter interaction.^70,91^

Assuming a monochromatic and perturbative CW excitation, the RIXS cross-section *I*_RIXS_ can be obtained from the time evolution of the second-order correction to the system wave function^92^
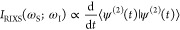
12Following Lee and Heller,^70,92^ the time-dependent second-order perturbation wave function is recursively defined by
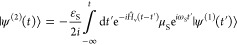
13with the first-order wave function
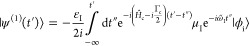
14where |ϕ_i_⟩ ≡ |ϕ_i_(−∞)⟩ is the initial unperturbed wave function with eigenenergy *E*_*i*_, μ_I_ and μ_S_ are the transition dipole moment operators along the direction of the incident and the scattering field, ε_I_ and ε_S_ are monochromatic with frequency  and , respectively, and where  is defined by . Furthermore, Γ_c_ denotes the intrinsic lifetime broadening of the core-excited states. Changing the variables τ = *t*′ – *t*″, the first-order wave function can be rewritten as
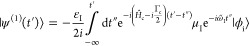
15
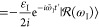
16with the Raman wave function  given by
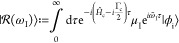
17

Although the Raman wave function is not an eigenfunction of either Hamiltonian involved, it forms a pseudo-time-independent intermediate state, which contains all dynamical information prior to the scattering event. Finally, inserting eqs 16 and 13 into eq 12 and including the inverse final state lifetime Γ_f_ leads to a time-dependent expression for the total RIXS spectrum
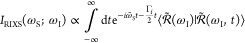
18where  and  is an evolving wavepacket on the ground and valence-excited state manifold. Furthermore,  defines the energy loss of the system.

Performing the time-integral in eq 18 yields the frequency-dependent form of the RIXS cross section
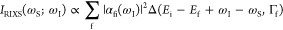
19where the scattering amplitude α_fi_ is governed by the well-known KHD formula
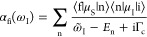
20with |i⟩, |n⟩, and |f⟩ being the initial, intermediate, and final vibronic eigenstates involved in the RIXS process with energies *E*_i_, *E*_n_, and *E*_f_, respectively. Furthermore, the Lorentzian line shape function
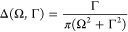
21with full width at half-maximum (fwhm) Γ causes a phenomenological broadening. The KHD expression thus provides a static perspective of the RIXS event, where the scattering amplitudes are dependent on the Franck–Condon overlaps of all relevant states as well as the amount of detuning  from each intermediate state.

Both, the time-dependent (eq 18) and time-independent (eq 19) expressions for the RIXS cross sections rely on CW conditions and thus neglect any contributions stemming from the spectral content of the incident radiation. In order to incorporate the spectral content required for a finite duration of the incident field, we go beyond the CW picture and assume an -shaped X-ray pulse as given in eq 3, describing a more realistic situation for time-resolved experiments. Without loss of generality, the pulse is centered at *t*_0_ = 0 and has the carrier frequency . The first-order wave function now reads
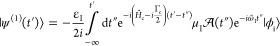
22while the second-order wave function is still recursively defined as given in eq 13. Using the Fourier representation of the envelope function
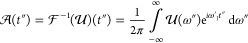
23and again changing the variables τ = *t*′ – *t*″, the first-order wave function can be rewritten as
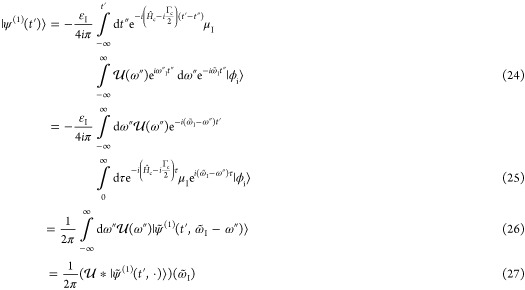
where * denotes the convolution and  is given by
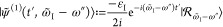
28Analogously to eqs 13 and 16, we recursively define  by
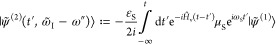
29yielding the following expression for the second-order wave function
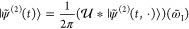
30since the convolution is linear. Within the spontaneous emission process, a transition from every eigenstate component of a wavepacket occurs independently, populating a different final state for large enough *t* (see Appendix A) and thus
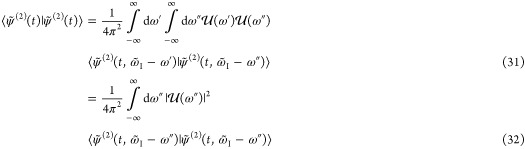
and use of eq 12 leads to
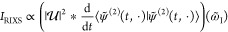
33

This dependence demonstrates that the impact of a limited pulse duration has two distinct aspects. First, when longitudinally coherent X-ray pulses with a wide spectral range are used, more resonances are encompassed within the coherent pulse excitation. This not only affects the finer vibronic substructure and peak intensity in the final spectra but also decreases the spectral sensitivity of the detuning effect. Additionally, the finite duration of the incident X-ray beam causes a general broadening of the RIXS spectra, given by the convolution with the squared absolute value of the Fourier transform of the incident field envelope function. Note that an incoherent spectral distribution as encountered at synchrotron radiation facilities is not described by a spectral convolution but rather a spectrally weighted average.

### Quantum Dynamics

**Figure 1:**
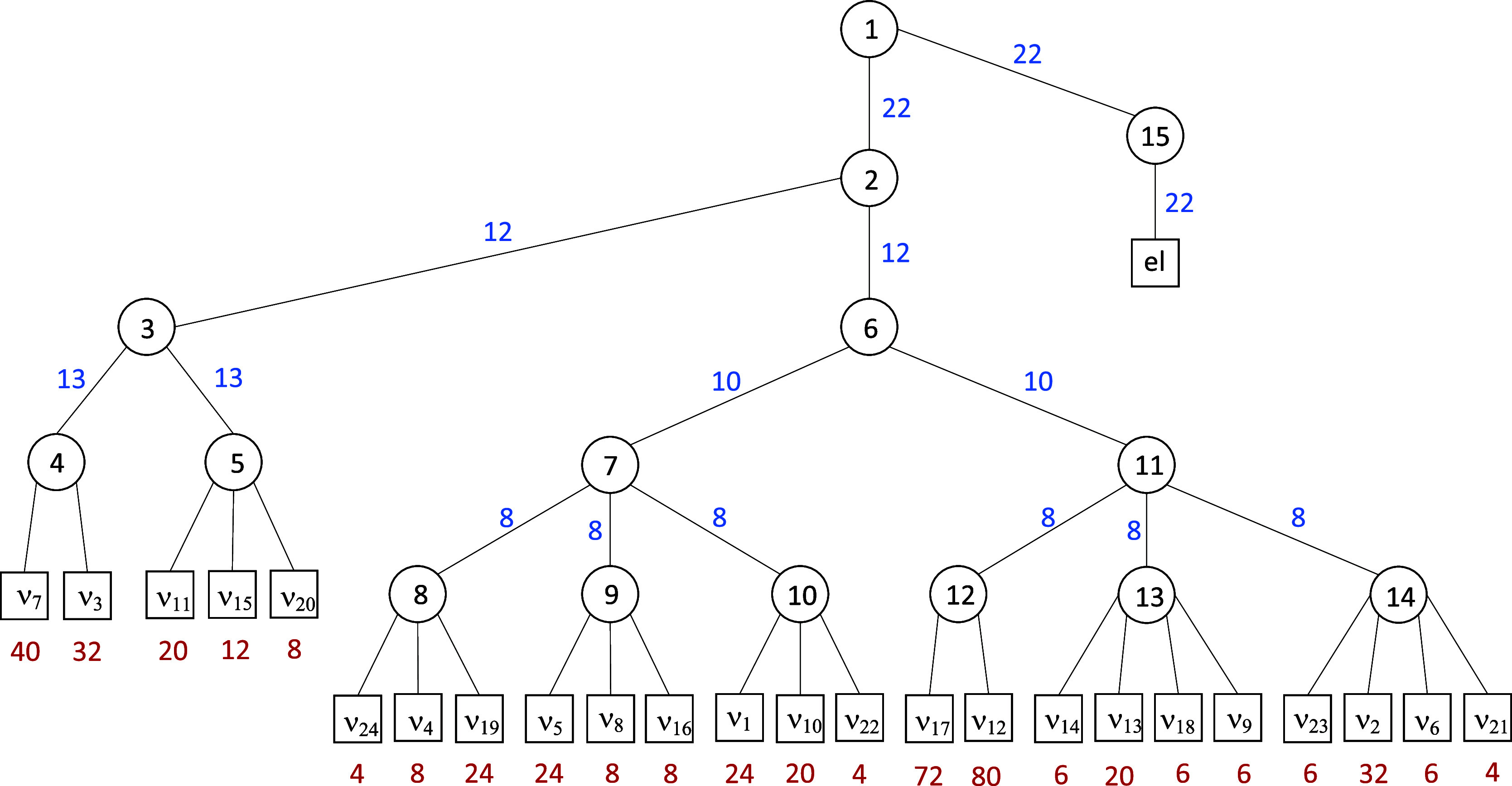
Tree structure of the ML-MCTDH simulations, including all 24 normal modes. The maximum depth of the tree is five layers where the first one separates the 24 vibrational coordinates and the discrete electronic degree of freedom in the single-set formulation. The circles represent the node in the layer structure, and the number *n* of SPFs (blue) is given next to the link lines. The last layer comprises the vibrational normal modes, where the number *N* of primitive functions (red) is used to represent the grid. All values are taken from ref (93).

The nuclear time-dependent Schrödinger equation was solved employing the Heidelberg implementation of the multilayer (ML-) MCTDH method.^93−96^ In this approach, MCTDH is recursively applied through different layers of effective modes, within which the equations of motion are determined from the Dirac–Frenkel variational principle.^97,98^ The layer structure as well as the discrete variable representation, number of grid points, and single-particle function (SPF) basis size were adapted from the ML-2 model in ref (93) without modifications except for the number of electronic states. In particular, this ML ansatz for a full dimensional wave function including all vibrational normal modes was proven to offer a reasonable balance of quality and computational effort. The details are reprinted in Figure 1 for completeness. Core-excited state propagations were run until the pseudo-time-independent intermediate state is reached while dynamics happening in the valence states were run for 100 fs. The data output was written every 0.1 fs, guaranteeing a sufficient time resolution and frequency span for subsequent Fourier transforms.

## Results and Discussion

### Parametrization of the Model Hamiltonian

Pyrazine (C_4_H_4_N_2_) belongs to point group *D*_2*h*_ at neutral ground-state equilibrium geometry and possesses 24 vibrational normal modes. The vibrational frequencies computed at the CCSD/aug-cc-pVDZ level of theory are listed in Table 1 along with their symmetry and a comparison to the experimental data.

**Table 1:** Harmonic Ground-State Vibrational Frequencies (in cm^–1^) Obtained at the CCSD/aug-cc-pVDZ Level along with the Experimental Data^99^^a^

mode	Wilson	symmetry	cm^–1^	Exp.
ν_1_	ν_16*a*_	*a*_*u*_	350	341
ν_2_	ν_16*b*_	*b*_3*u*_	425	420
ν_3_	ν_6*a*_	*a*_*g*_	604	596
ν_4_	ν_6*b*_	*b*_3*g*_	709	704
ν_5_	ν_4_	*b*_2*g*_	737	756
ν_6_	ν_11_	*b*_3*u*_	805	785
ν_7_	ν_10*a*_	*b*_1*g*_	943	919
ν_8_	ν_5_	*b*_2*g*_	950	983
ν_9_	ν_17*a*_	*a*_*u*_	980	960
ν_10_	ν_12_	*b*_1*u*_	1033	1021
ν_11_	ν_1_	*a*_*g*_	1038	1015
ν_12_	ν_18*b*_	*b*_2*u*_	1091	1063
ν_13_	ν_14_	*b*_2*u*_	1146	1149
ν_14_	ν_18*a*_	*b*_1*u*_	1162	1136
ν_15_	ν_9*a*_	*a*_*g*_	1253	1230
ν_16_	ν_3_	*b*_3*g*_	1367	1346
ν_17_	ν_19*b*_	*b*_2*u*_	1441	1416
ν_18_	ν_19*a*_	*b*_1*u*_	1518	1484
ν_19_	ν_8*b*_	*b*_3*g*_	1595	1525
ν_20_	ν_8*a*_	*a*_*g*_	1650	1582
ν_21_	ν_7*b*_	*b*_3*u*_	3196	3040
ν_22_	ν_13_	*b*_1*u*_	3197	3012
ν_23_	ν_20*b*_	*b*_2*u*_	3213	3063
ν_24_	ν_2_	*a*_*g*_	3218	3055

a The modes are labeled by ascending frequency and compared to Wilson’s notation.^100^

**Figure 2:**
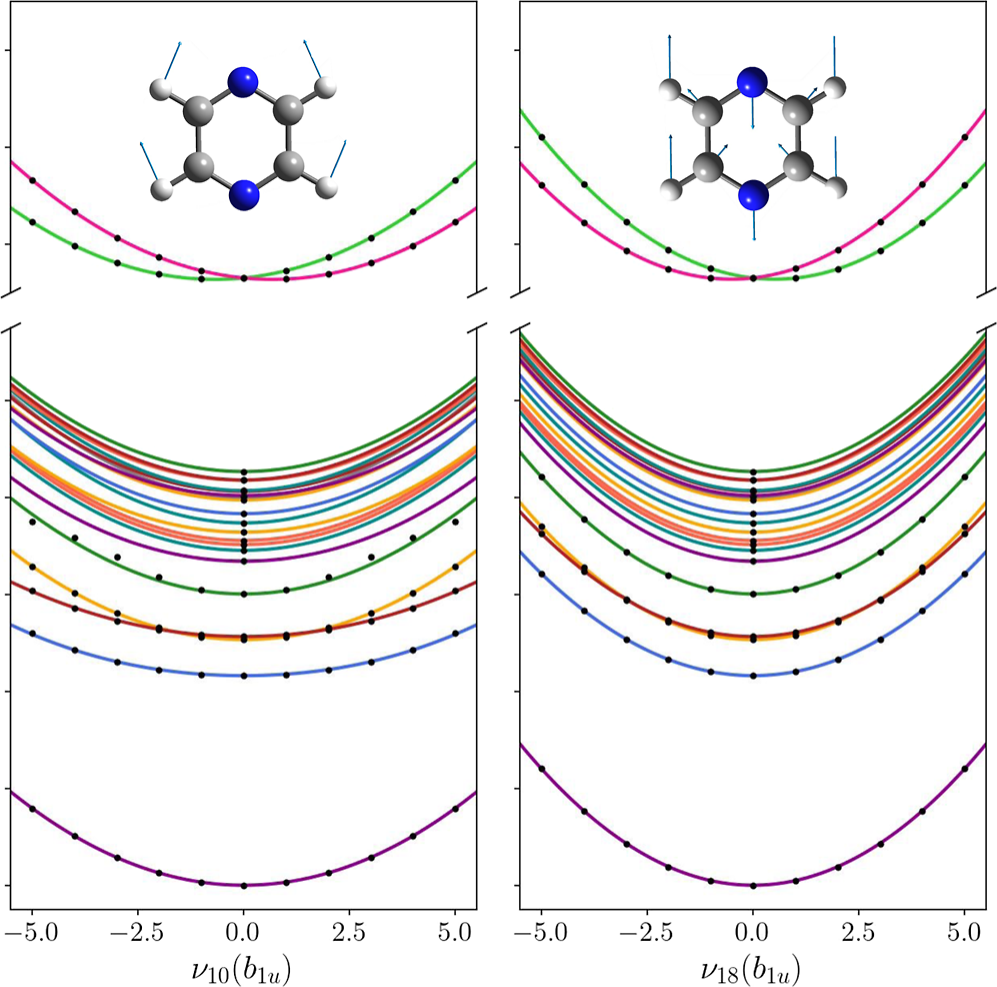
Cuts through the diabatic PESs along the ν_10_(*b*_1*u*_) and ν_18_(*b*_1*u*_) vibrational normal modes that mainly drive the symmetry breaking in the core-excited states. Label and symmetry for each state can be found in Table 2. The adiabatic energies (black points) are obtained from ab initio calculations using the (fc-CVS-) EOM-CCSD/aug-cc-pVDZ method.

The diabatic Hamiltonian **H**_mol_ comprises two sub-Hamiltonian **H**_v_ and **H**_c_, accounting for the valence- and core-excited states dynamics, respectively. **H**_c_ involves two core-excited states that are nearly degenerate at the Franck–Condon point. In the vicinity of that point, the next energetically higher electronic states are well separated and can hence be disregarded. The four vibrational modes with symmetry *B*_1*u*_ are potential candidates to vibronically couple the two nearly degenerate core-excited states where the conical intersection formed along these modes takes place exactly at the Franck–Condon point. Cuts of the PESs along the two strongest coupling modes, ν_10_(*B*_1*u*_) and ν_18_(*B*_1*u*_), are shown in Figure 2. The lifetime of the core-excited states is assumed to be 8 fs^101^ captured by an imaginary energy term in the core Hamiltonian **H**_c_.

Since RIXS has the ability to reach higher-lying valence-excited states, we include 20 electronic states in **H**_v_. These states consist of the ground state and the 19 energetically lowest valence-excited singlet states, covering an approximate spectral range of 10 eV. The computed vertical excitation energies and symmetries for all electronic states are listed in Table 2. It is worth noting that the ordering of very closely lying states can differ between different levels of theory.^102,103^ This concerns not only *S*_2_(*B*_2*u*_) and *S*_3_(*A*_*u*_) but also the region with a high density of states. To maintain a consistent model within this study, we treat every valence-excited state at the same level of theory without any adjustment of the vertical excitation energy.

**Table 2:** State Symmetries and Vertical Excitation Energies *E*^(α)^ (in eV) at the Franck–Condon Point of All Electronic States Considered in This Work^a^

state	symmetry	*E*^(α)^
S_0_	*A*_*g*_	0.00
S_1_	*B*_3*u*_	4.32
S_2_	*B*_2*u*_	5.07
S_3_	*A*_*u*_	5.13
S_4_	*B*_2*g*_	6.01
S_5_	*A*_*g*_	6.68
S_6_	*B*_1*u*_	6.91
S_7_	*B*_1*g*_	7.02
S_8_	*B*_1*g*_	7.11
S_9_	*B*_2*u*_	7.28
S_10_	*B*_1*u*_	7.47
S_11_	*B*_3*u*_	7.66
S_12_	*B*_2*u*_	7.94
S_13_	*B*_3*g*_	8.00
S_14_	*A*_*g*_	8.04
S_15_	*A*_*u*_	8.12
S_16_	*B*_1*u*_	8.14
S_17_	*B*_1*g*_	8.35
S_18_	*A*_*u*_	8.36
S_19_	*B*_2*g*_	8.53
X_1_	*B*_2*g*_	402.30
X_2_	*B*_3*u*_	402.30

a Valence-excited state energies were evaluated using EOM-CCSD while core-excited state energies were obtained using (fc-CVS-) EOM-CCSD. In both cases, the aug-cc-pVDZ basis set was used.

Both sub-Hamiltonians, **H**_v_ and **H**_c_, were then approximated by a vibronic coupling model including all vibrational degrees of freedom. The highest frequency mode ν_24_ was fitted by Morse potentials to account for anharmonicity. Since the density of valence-excited states above 6 eV increases rapidly, we used the linear intra- and interstate coupling constants as obtained from EOM-CCSD/aug-cc-pvDZ calculations at the Franck–Condon point to parametrize these electronic states (S_5_ – S_19_). The linear coupling parameters, which mainly drive the core-excited state dynamics at short times, are listed in Table 3. A complete list of all parameters is further provided in the Supporting Information.

**Table 3:** Linear Intra- and Interstate Coupling Constants κ_i_^(n)^ and λ_i_^(nm)^, Respectively, for the Core-Excited States, X_*n*_, Obtained in This Work

	κ_3_	κ_11_	κ_15_	κ_20_
X_1_	0.02738	–0.04034	0.05568	0.10433
X_2_	0.02627	–0.04050	0.05619	0.10457

	λ_10_	λ_14_	λ_18_	κ_22_
(X_1_, X_2_)	0.08680	0.01326	0.09910	0.03014

The transition dipole moments and oscillator strengths for each pair of valence- and core-excited states were computed at the Franck–Condon point using the same level of theory. We neglect transitions where the transition dipole moment was below 0.01 and thus less than 10% of the strongest core–valence transition. The corresponding transition dipole moments are listed in Table 4.

**Table 4:** Transition Dipole Moments μ_fi_ between States i and f Obtained from fc-CVS-EOM-CCSD/aug-cc-pVDZ Calculations

state transition	μ_fi_
X_2_ ← S_0_	0.10
X_1_ ← S_1_	0.06
X_2_ ← S_4_	0.06
X_1_ ← S_6_	0.02
X_1_ ← S_16_	0.04
X_1_ ← S_18_	0.04

### Symmetry Distortion Induced by Core-Excited State Dynamics

**Figure 3:**
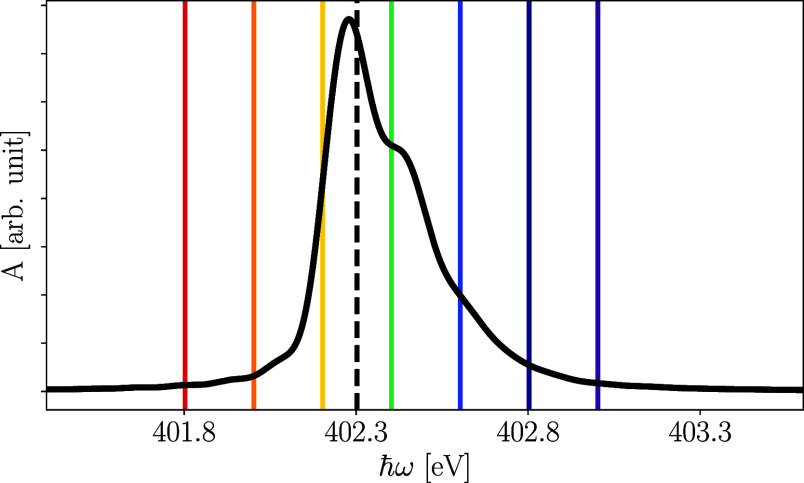
Computed static X-ray absorption spectrum of pyrazine at the nitrogen K-edge. The vertical lines indicate the excitation energies used to simulate the RIXS spectra. Here, the energy related to the dashed black line corresponds to the vertical excitation energy of X_2_ used to calculate the RIXS spectrum shown in this section. The energies indicated by the colored lines relate to the detuning effect discussed in the next section.

We consider the scattering event initiated by the resonant absorption of an X-ray photon, which promotes an electron from a nitrogen (N-)1s orbital to the lowest unoccupied molecular orbital (LUMO). Pyrazine contains two equivalent N atoms, so a linear combination of both N-1s orbitals builds symmetric and antisymmetric core-orbitals that are nearly degenerate. Excitation from these core-orbitals leads in turn to two nearly degenerate core-excited states, X_1_(*B*_2*g*_) and X_2_(*B*_3*u*_). Although only transitions from the antisymmetric core orbitals are allowed by symmetry, both core-excited states must be considered to account for nonadiabatic transitions induced by vibronic coupling. Figure 3 shows the static X-ray absorption spectrum, resulting from an electronic transition between the antisymmetric core orbital and the LUMO. The absorption spectrum was obtained from the Fourier transform of the autocorrelation function of the dipole-operated ground state, thus equivalent to a δ-pulse excitation.^92,104,105^

**Figure 4:**
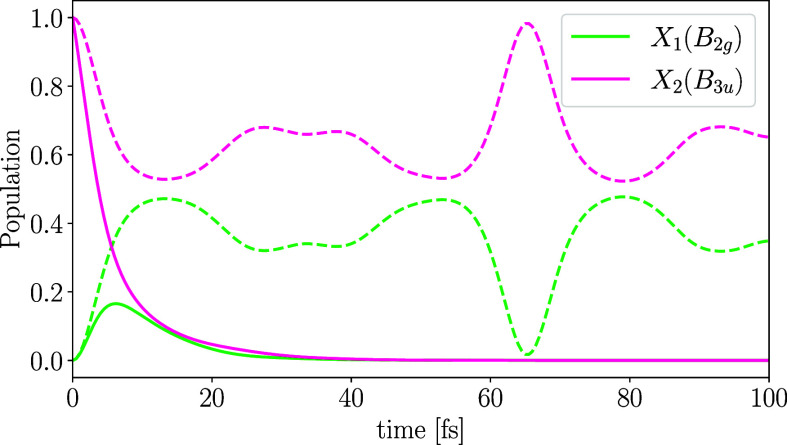
Core-excited state populations of the diabatic X_1_(*B*_2*g*_) (green) and X_2_(*B*_3*u*_) (magenta) states starting from X_2_(*B*_3*u*_) at the Franck–Condon point. The solid lines present the total diabatic state population including the core-hole decay while the dashed lines correspond to the normalized diabatic state population.

The related diabatic population transfer is illustrated in Figure 4. After an instantaneous, vertical excitation at time 0, the bright X_2_(*B*_3*u*_) state rapidly depopulates into the dark X_1_(*B*_2*g*_) state, leading to an almost equal distribution of population between the two states within the short core-hole lifetime. This ultrafast, nonadiabatic population transfer can be traced back to the symmetry-allowed conical intersection between these states, which is formed in the immediate vicinity of the Franck–Condon point (see Figure 2) and primarily driven by the two asymmetric normal modes ν_10_ and ν_18_. These dynamics result in a final localization of the core-hole.

**Figure 5:**
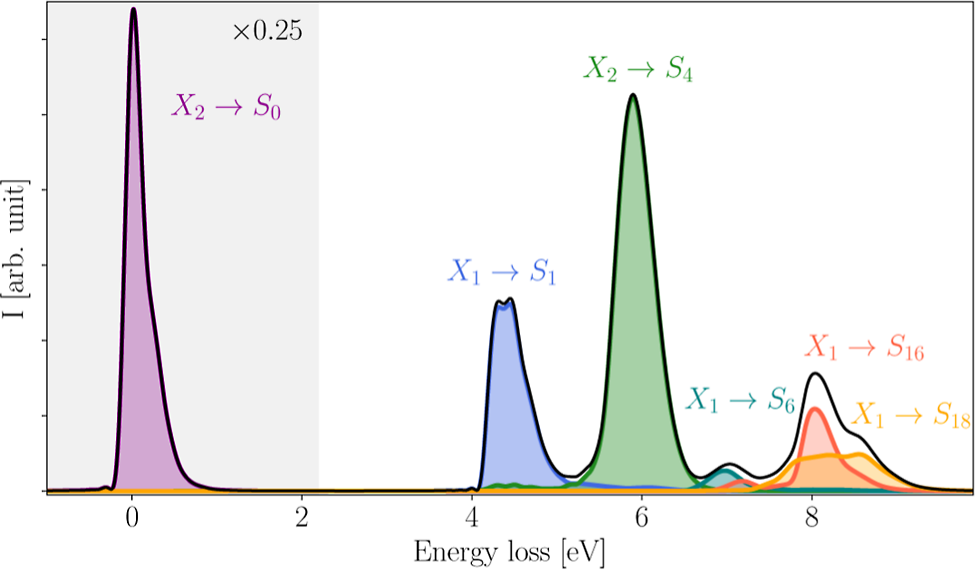
Simulated RIXS spectrum of pyrazine at the nitrogen K-edge. The contributions of the transitions to S_0_, S_1_, S_4_, S_6_, S_16_, and S_18_ to the total spectrum are highlighted in purple, blue, green, cyan, red, and orange, respectively. The elastic peak (purple) is downscaled to 25% for better visualization. Before performing the Fourier transformation in eq 18, the window function  was applied to reduce the Gibbs phenomenon. Here, we assume a damping time of *T* = 30 fs in order to include broadening caused by dephasing mechanisms.

The impact of symmetry breaking induced by core-excited state dynamics is clearly seen in the RIXS spectrum of pyrazine at the nitrogen K-edge presented in Figure 5. The spectrum was obtained using eq 18, assuming a monochromatic X-ray photon beam with carrier frequency equal to the vertical excitation energy from the ground state to the excited state X_2_. It displays five prominent bands arising from six electronic transitions. The asymmetric shape of the elastic peak already suggests that the system undergoes nuclear displacements on the core-excited state manifold. As expected from the transition dipole moments (see Table 4), the elastic peak is the dominant feature of the spectrum but without obscuring any contributions from inelastic transition due to the significant energy gap between the ground and valence-excited states. Moreover, the spectrum reveals four bands of inelastic electronic transitions located at approximately 4.5, 6.0, 7.0, 8.1, and 8.6 eV where the latter two exhibit a large spectral overlap and are thus barely distinguishable in the overall spectrum. While the emission band located at 6.0 eV is attributable to the electronic X_2_ → S_4_ transition, the other four bands originate from population of the optically dark X_1_(*B*_2*g*_) state which can only be accessed through nonadiabatic population transfer from the bright X_2_(*B*_3*u*_) state. Consequently, the symmetry breaking caused by ultrafast core-excited state dynamics allows for four additional electronic transitions that would otherwise be forbidden for two consecutive dipole transitions (i.e., by quadrupole selection rules) within this spectral range.

### Dynamical Control by Detuning

Dynamical processes in the core-excited states can be controlled by detuning the frequency of the incident radiation that is used to prepare the intermediate state of the system. The effective scattering duration in RIXS is determined by the time the wavepacket spends in the intermediate core-excited states, i.e., the time interval [0, τ] that contributes to the Raman wave function (eq 17). Due to the uncertainty relation for the energy, the propagation time shortens the further the excitation energy is from resonance and is given by^106^
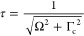
34where Ω is the amount of detuning and Γ_c_ inversely determines the core-hole lifetime.

**Figure 6:**
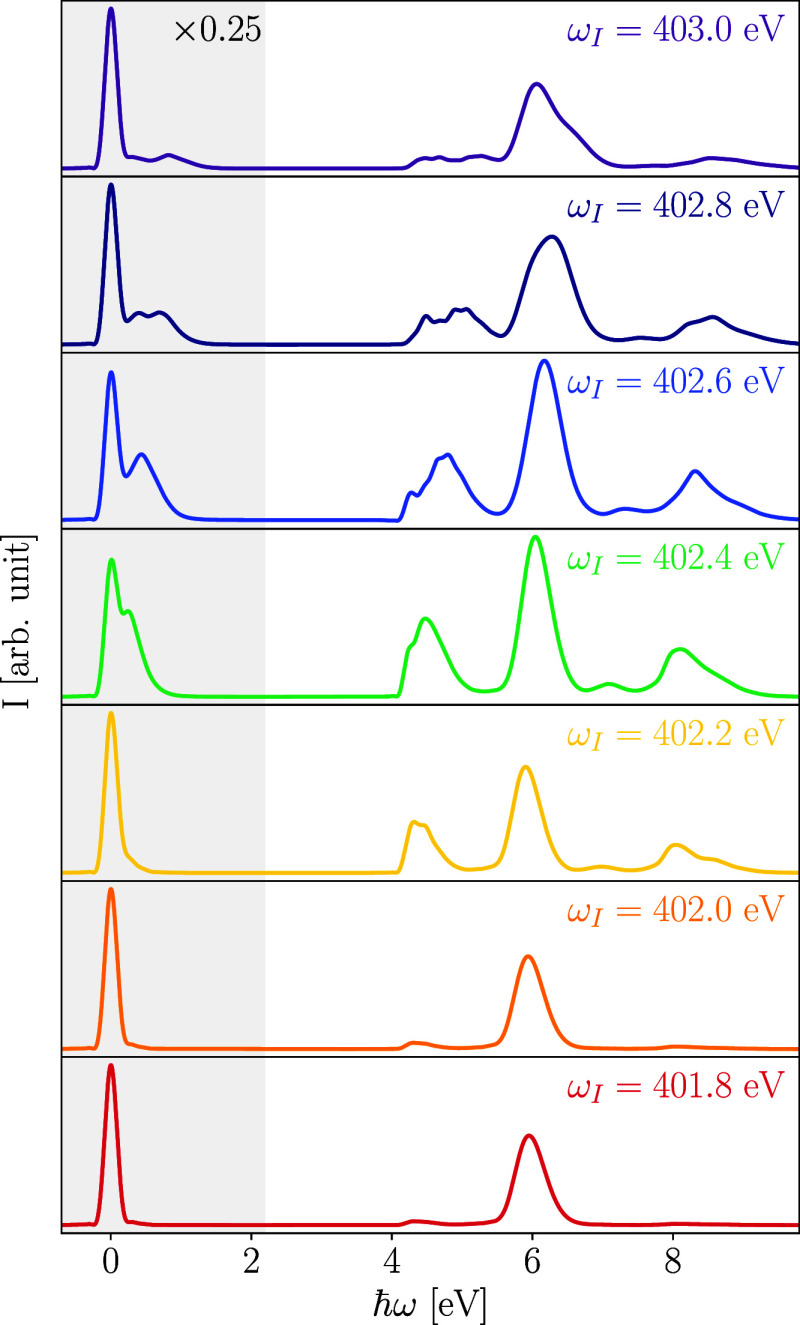
Dependence of the RIXS profile on the incident photon energy. All spectra are independently normalized to their respective elastic peak height before rescaling the signals in the gray spectral region to 25%. The energies of the incident radiation used for these calculation are also highlighted in the photoabsorption band, as shown in Figure 3.

The dependence of the RIXS signal for pyrazine on the incoming photon frequency, ω_I_, is shown in Figure 6. The four inelastic emission channels stemming from the dark X_1_(*B*_2*g*_) state exhibit a strongly detuning-dependent intensity. In particular, when far from resonance, i.e., for incoming excitation energies below ∼402.0 eV, these bands almost disappear. Moreover, in this region, the elastic peak exhibits a symmetric Lorentzian line shape indicative of a single transition. Both observations show that the effective scattering duration becomes so short for large detuning that dynamical effects are negligible. In contrast, for excitation energies greater than 402.0 eV, the signature of vibrational progressions is apparent on the positive energy side of the elastic peak, and inelastic Raman transitions stemming from both core-excited states, X_1_ and X_2_, are prominent. Their shapes differ for different excitation energies, reflecting the nuclear motion in the core-excited states. In particular, for excitation energies greater than the vertical excitation energy, a rather broad and asymmetrical shape can be observed for each loss peak.

### Spectral Distribution due to Finite Pulse Duration

While the previous sections are based on light–matter interaction with monochromatic plane X-ray waves, in the following section, we investigate the influence of the incident X-ray spectrum on the resolution of the RIXS spectra. The RIXS cross sections are calculated using eq 33 where the external electric field was given by a normalized Gaussian-shaped X-ray pulse, i.e., the pulse envelope function is
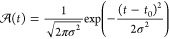
35where *t*_0_ denotes the pulse center and the standard deviation σ is linked to the temporal fwhm duration *F*_t_ by . According to eq 33, a spectral broadening of the RIXS spectrum is caused by the convolution with the square of the absolute value of the Fourier transform of the envelope function. For an envelope function of the form in eq 35, this is again a Gaussian function
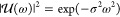
36with spectral fwhm . In particular, the fwhm ratio is here *F*_t_·*F*_ω_ ≈ 3.921.

**Figure 7:**
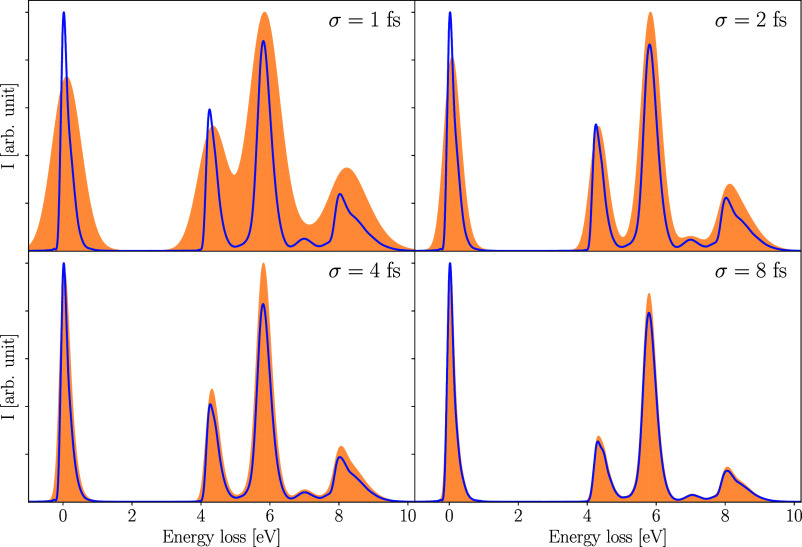
Dependence of RIXS signals on the duration of the incident radiation field. Each spectrum was derived using eq 33 (orange shadow) and eq 37 (blue line), where a Gaussian X-ray pulse was used to trigger the core-excited state dynamics. The carrier frequency was kept fixed while the temporal standard deviation varies from 1 to 8 fs (equivalent to Γ_c_/8 to Γ_c_). The same window function as that in Figure 5 was applied before performing the Fourier transformation.

Due to the reciprocal connection between temporal and spectral width, shorter pulses create spectrally broader wavepackets and vice versa, leading to different signatures of the RIXS signal. This aspect is described by the term
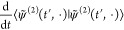
37in eq 33 and can be considered independently from the general broadening of the signal described by the convolution with  although both aspects have the same origin. Figure 7 illustrates both effects on the RIXS spectra of pyrazine for different incoming X-ray pulses where the temporal duration was varied, but the carrier frequency of the pulse was kept fixed at 402.3 eV. The spectra shown in blue are evaluated in accordance with eq 37, while the orange shadowed spectra represent the total cross section as obtained from eq 33.

In general, the different RIXS spectra are in good agreement. However, the relative intensities vary depending on the length of the X-ray pulse. Specifically, emission channels starting from the dark X_1_ at around 6.0, 7.0, and 8.4 eV appear to be more likely for shorter pulse duration. Furthermore, the shape of the spectral lines changes slightly depending on the distribution of the field. This is evident in the X_1_ → S_1_ emission band, where distinct progressions become more prominent with longer pulse duration.

While for pulses with FWHM of about a third of the dephasing time of the valence excitations or longer, the broadening resulting from the spectral distribution of the field is less significant compared to other dephasing effects, shorter pulses cause a more pronounced broadening of the signal, as shown by the orange shadow. This effect should hence be considered for optimal spectral and temporal resolution as well as the detuning capability. Especially for very short pulses, the finer details of the vibronic structure in the emission bands are almost completely suppressed, which can complicate the analysis of core-excited state dynamics when the effectiveness of detuning as a control mechanism is also reduced.

## Conclusions

In this study, we benchmarked a fully time-dependent approach within the MCTDH framework to simulate RIXS spectra. We used pyrazine as a model system to examine this resonant scattering process at the nitrogen K-edge, considering wavepacket dynamics in all of the electronic states of the model. Our findings reveal ultrafast symmetry breaking of the molecule in the core-excited states, leading to a significant alteration of the symmetry selection rules. This behavior underscores the crucial role of non-adiabatic nuclear core-excited state dynamics for the correct description of RIXS signals. Furthermore, we explored the detuning dependence of the RIXS spectra in our time-dependent approach. Our results highlight the importance of detuning as a control mechanism for ultrafast dynamical processes, which allows for predicting not only the intensity of both inelastic and elastic emission channels but also the vibrational substructure of individual electronic transitions. Additionally, we investigated how the duration of a coherent X-ray pulse affects the RIXS spectrum.

In summary, we have described RIXS as a fully time-dependent process including electronic and nuclear dynamics in all core- and valence-excited states of the model and with a temporally arbitrary incident X-ray field. Our work demonstrates how the spectral distribution of the X-ray pulse impacts the individual RIXS pathways, and how it leads to a general broadening effect of the loss spectra. This research provides insights into the intricacies of inelastic X-ray scattering in the presence of core-hole symmetry breaking, under detuning, and for excitations with coherent X-ray pulses on the order of or shorter than the core-hole lifetime. Furthermore, our time-dependent simulation approach can be used for modeling ultrafast RIXS experiments at pulsed X-ray sources such as free-electron lasers.

## Appendix A

### Emission from a Wavepacket

In order to derive an expression for the probability that a photon with energy ℏω is emitted from a wavepacket, we consider a system containing two electronic states. The total Hamiltonian reads

38

39

where the Hamiltonian  describes the unperturbed system and the interaction Hamiltonian  is treated as perturbation. Here, the molecular Hamiltonian  of the two-state system is given by

40

where *E*_n_ and |n⟩, n ∈ {i, f}, are the eigenenergies and eigenstates, respectively, of the initial and final electronic state. The light Hamiltonian  is further defined through

41

with the vacuum state |vac⟩ where we ignore directions and polarizations for simplicity. For an interaction operator  between the molecule and photon, the light–matter interaction term can be written as

42

43

Assuming a wavepacket has been initially created as a superposition of the initial electronic state, i.e.,

44

the transition rate Γ_ω_ of emitting photon with energy ℏω is defined by

45

where the transition probability *P*_ω_(*t*)≔|⟨ω|ψ(*t*)⟩|^2^ can be evaluated using first-order perturbation theory

46

where |ψ^(0)^(0)⟩ = |ψ(0)⟩ and |ψ^(1)^(0)⟩ = 0. Considering only induced transitions between two different states, the probability then reads

47

where the first-order correction ψ^(1)^ to the wavefunction of the system ψ is defined by

48

with

49

50

51

52

As ∫dω⟨ω′|ω⟩ = ∫dωδ(ω′ – ω) = 1, it follows

53

54

55

56

where Ω_if_≔ω_i_ – ω_f_. Hence, the emission probability per unit time in first-order correction is given by

57

58

59

For Ω_if_ ≠ ω and Ω_jf_ ≠ ω, it holds

60

and in the same way

61

Therefore, it follows for the product of a zero and bounded sequence

62

and the transition rate Γ_ω_ thus vanishes for Ω_if_, Ω_jf_ ≠ ω.

Let now Ω_if_ ≠ ω but Ω_jf_ = ω. Applying the rule of l’Hopital

63

we can show that the fraction above is purely imaginary. Hence, it follows

64

65

66

67

68

and thus again Γ_ω_ → 0 for *t* → ∞. Analogously, Γ_ω_ is zero for Ω_if_ = ω and Ω_jf_ ≠ ω. Hence, only the case Ω_if_ = ω and Ω_jf_ = ω, so in particular *i* = *j*, contributes to the transition rate. Starting from eq 59, we get

69

70

71

72

73

74

where |i_γ_(*t*)⟩ denotes the propagation of the projected eigenstate |i⟩ onto the final excited state manifold. So, every eigenstate from the wavepacket emits independently, where the contribution of a certain eigenstate is determined by the evolution of the projected wavepacket |i_γ_(*t*)⟩ weighted by the initial population of state |i⟩. In particular, the spontaneous emission from a wavepacket is incoherent, in the sense that every transition from this wavepacket populates a different and hence orthogonal final state of the material system and the electromagnetic field.

## Supporting Information Available

On-diagonal linear intrastate coupling constants κ_i_^(n)^, off-diagonal linear interstate coupling constants λ_i_^(nm)^, and on-diagonal bilinear (quadratic) coupling constants γ_i_^(n)^ and the parameters concerning the Morse potential (PDF)

## References

[ref1] Tavares P. F.; Leemann S. C.; Sjöström M.; Andersson Å. The MAXIV storage ring project. J. Synchrotron Radiat. 2014, 21, 862–877. 10.1107/S1600577514011503. 25177978 PMC4181638

[ref2] Willmott P. An Introduction to Synchrotron Radiation: Techniques and Applications; Wiley: Hoboken, 2019.

[ref3] Willmott P. R.. In Magnetism and Accelerator-Based Light Sources; Bulou H., Joly L., Mariot J.-M., Scheurer F., Eds.; Springer International Publishing: Cham, 2021, pp 1–37.

[ref4] McNeil B. W. J.; Thompson N. R. X-ray free-electron lasers. Nat. Photonics 2010, 4, 814–821. 10.1038/nphoton.2010.239.

[ref5] Pellegrini C.; Marinelli A.; Reiche S. The physics of x-ray free-electron lasers. Rev. Mod. Phys. 2016, 88, 015006 10.1103/RevModPhys.88.015006.

[ref6] Zhao Z.; Wang D.; Gu Q.; Yin L.; Gu M.; Leng Y.; Liu B. Status of the SXFEL Facility. Appl. Sci. 2017, 7, 607 10.3390/app7060607.

[ref7] Seddon E. A.; Clarke J. A.; Dunning D. J.; Masciovecchio C.; Milne C. J.; Parmigiani F.; Rugg D.; Spence J. C. H.; Thompson N. R.; Ueda K.; Vinko S. M.; Wark J. S.; Wurth W. Short-wavelength free-electron laser sources and science: a review. Rep. Prog. Phys. 2017, 80, 115901 10.1088/1361-6633/aa7cca. 29059048

[ref8] Ament L. J. P.; van Veenendaal M.; Devereaux T. P.; Hill J. P.; van den Brink J. Resonant inelastic X-ray scattering studies of elementary excitations. Rev. Mod. Phys. 2011, 83, 705–767. 10.1103/RevModPhys.83.705.

[ref9] Zhang Y.; Biggs J. D.; Healion D.; Govind N.; Mukamel S. Core and valence excitations in resonant X-ray spectroscopy using restricted excitation window time-dependent density functional theory. J. Chem. Phys. 2012, 137, 194306 10.1063/1.4766356. 23181305 PMC3517496

[ref10] Beye M.; Wernet P.; Schüßler-Langeheine C.; Föhlisch A. Time resolved resonant inelastic X-ray scattering: A supreme tool to understand dynamics in solids and molecules. J. Electron Spectrosc. Relat. Phenom. 2013, 188, 172–182. 10.1016/j.elspec.2013.04.013.

[ref11] Mukamel S.; Healion D.; Zhang Y.; Biggs J. D. Multidimensional Attosecond Resonant X-Ray Spectroscopy of Molecules: Lessons from the Optical Regime. Annu. Rev. Phys. Chem. 2013, 64, 101–127. 10.1146/annurev-physchem-040412-110021. 23245522 PMC3721744

[ref12] Wernet P.; Kunnus K.; Josefsson I.; Rajkovic I.; Quevedo W.; Beye M.; Schreck S.; Grübel S.; Scholz M.; Nordlund D.; Zhang W.; Hartsock R. W.; Schlotter W. F.; Turner J. J.; Kennedy B.; Hennies F.; de Groot F. M. F.; Gaffney K. J.; Techert S.; Odelius M.; Föhlisch A. Orbital-specific mapping of the ligand exchange dynamics of Fe(CO)_5_ in solution. Nature 2015, 520, 78–81. 10.1038/nature14296. 25832405

[ref13] Eckert S.; Norell J.; Miedema P. S.; Beye M.; Fondell M.; Quevedo W.; Kennedy B.; Hantschmann M.; Pietzsch A.; Van Kuiken B. E.; Ross M.; Minitti M. P.; Moeller S. P.; Schlotter W. F.; Khalil M.; Odelius M.; Föhlisch A. Ultrafast Independent N-H and N-C Bond Deformation Investigated with Resonant Inelastic X-Ray Scattering. Angew. Chem., Int. Ed. 2017, 56, 6088–6092. 10.1002/anie.201700239. PMC5485001 28374523

[ref14] Jay R. M.; Norell J.; Eckert S.; Hantschmann M.; Beye M.; Kennedy B.; Quevedo W.; Schlotter W. F.; Dakovski G. L.; Minitti M. P.; Hoffmann M. C.; Mitra A.; Moeller S. P.; Nordlund D.; Zhang W.; Liang H. W.; Kunnus K.; Kubiček K.; Techert S. A.; Lundberg M.; Wernet P.; Gaffney K.; Odelius M.; Föhlisch A. Disentangling Transient Charge Density and Metal–Ligand Covalency in Photoexcited Ferricyanide with Femtosecond Resonant Inelastic Soft X-ray Scattering. J. Phys. Chem. Lett. 2018, 9, 3538–3543. 10.1021/acs.jpclett.8b01429. 29888918

[ref15] Lu H.; Gauthier A.; Hepting M.; Tremsin A. S.; Reid A. H.; Kirchmann P. S.; Shen Z. X.; Devereaux T. P.; Shao Y. C.; Feng X.; Coslovich G.; Hussain Z.; Dakovski G. L.; Chuang Y. D.; Lee W. S. Time-resolved RIXS experiment with pulse-by-pulse parallel readout data collection using X-ray free electron laser. Sci. Rep. 2020, 10, 22226 10.1038/s41598-020-79210-4. 33335197 PMC7746750

[ref16] Chergui M.; Beye M.; Mukamel S.; Svetina C.; Masciovecchio C. Progress and prospects in nonlinear extreme-ultraviolet and X-ray optics and spectroscopy. Nat. Rev. Phys. 2023, 5, 578–596. 10.1038/s42254-023-00643-7.

[ref17] Monney C.; Patthey L.; Razzoli E.; Schmitt T. Static and time-resolved resonant inelastic X-ray scattering: Recent results and future prospects. X-Ray Spectrom. 2023, 52, 216–225. 10.1002/xrs.3311.

[ref18] Gel’mukhanov F.; Ågren H. Resonant X-ray Raman scattering. Phys. Rep. 1999, 312, 87–330. 10.1016/S0370-1573(99)00003-4.

[ref19] Ochmann M.; Vaz da Cruz V.; Eckert S.; Huse N.; Föhlisch A. R-Group stabilization in methylated formamides observed by resonant inelastic X-ray scattering. Chem. Commun. 2022, 58, 8834–8837. 10.1039/D2CC00053A. PMC9350990 35848855

[ref20] Harada Y.; Takeuchi T.; Kino H.; Fukushima A.; Takakura K.; Hieda K.; Nakao A.; Shin S.; Fukuyama H. Electronic Structure of DNA Nucleobases and Their Dinucleotides Explored by Soft X-ray Spectroscopy. J. Phys. Chem. A 2006, 110, 13227–13231. 10.1021/jp062720j. 17149838

[ref21] Weinhardt L.; Ertan E.; Iannuzzi M.; Weigand M.; Fuchs O.; Bär M.; Blum M.; Denlinger J. D.; Yang W.; Umbach E.; Odelius M.; Heske C. Probing hydrogen bonding orbitals: resonant inelastic soft X-ray scattering of aqueous NH_3_. Phys. Chem. Chem. Phys. 2015, 17, 27145–27153. 10.1039/C5CP04898B. 26417728

[ref22] Hussain A.; Huse N.; Vendrell O. Sensitivity of core-level spectroscopy to electrostatic environments of nitrile groups: An ab initio study. Struct. Dyn. 2017, 4, 054102 10.1063/1.5003404. 28966931 PMC5612798

[ref23] van Schooneveld M. M.; Gosselink R. W.; Eggenhuisen T. M.; Al Samarai M.; Monney C.; Zhou K. J.; Schmitt T.; de Groot F. M. F. A Multispectroscopic Study of 3 d Orbitals in Cobalt Carboxylates: The High Sensitivity of 2p3d Resonant X-ray Emission Spectroscopy to the Ligand Field. Angew. Chem., Int. Ed. 2013, 52, 1170–1174. 10.1002/anie.201204855. PMC3564409 23225760

[ref24] Wernet P.; Kunnus K.; Schreck S.; Quevedo W.; Kurian R.; Techert S.; de Groot F. M. F.; Odelius M.; Föhlisch A. Dissecting Local Atomic and Intermolecular Interactions of Transition-Metal Ions in Solution with Selective X-ray Spectroscopy. J. Phys. Chem. Lett. 2012, 3, 3448–3453. 10.1021/jz301486u. 26290971

[ref25] Kunnus K.; Zhang W.; Delcey M. G.; Pinjari R. V.; Miedema P. S.; Schreck S.; Quevedo W.; Schröder H.; Föhlisch A.; Gaffney K. J.; Lundberg M.; Odelius M.; Wernet P. Viewing the Valence Electronic Structure of Ferric and Ferrous Hexacyanide in Solution from the Fe and Cyanide Perspectives. J. Phys. Chem. B 2016, 120, 7182–7194. 10.1021/acs.jpcb.6b04751. 27380541

[ref26] Hahn A. W.; Van Kuiken B. E.; Chilkuri V. G.; Levin N.; Bill E.; Weyhermüller T.; Nicolaou A.; Miyawaki J.; Harada Y.; DeBeer S. Probing the Valence Electronic Structure of Low-Spin Ferrous and Ferric Complexes Using 2p3d Resonant Inelastic X-ray Scattering (RIXS). Inorg. Chem. 2018, 57, 9515–9530. 10.1021/acs.inorgchem.8b01550. 30044087

[ref27] Jay R. M.; Eckert S.; Fondell M.; Miedema P. S.; Norell J.; Pietzsch A.; Quevedo W.; Niskanen J.; Kunnus K.; Föhlisch A. The nature of frontier orbitals under systematic ligand exchange in (pseudo-)octahedral Fe(II) complexes. Phys. Chem. Chem. Phys. 2018, 20, 27745–27751. 10.1039/C8CP04341H. 30211412 PMC6240897

[ref28] Jay R. M.; Eckert S.; Van Kuiken B. E.; Ochmann M.; Hantschmann M.; Cordones A. A.; Cho H.; Hong K.; Ma R.; Lee J. H.; Dakovski G. L.; Turner J. J.; Minitti M. P.; Quevedo W.; Pietzsch A.; Beye M.; Kim T. K.; Schoenlein R. W.; Wernet P.; Föhlisch A.; Huse N. Following Metal-to-Ligand Charge-Transfer Dynamics with Ligand and Spin Specificity Using Femtosecond Resonant Inelastic X-ray Scattering at the Nitrogen K-Edge. J. Phys. Chem. Lett. 2021, 12, 6676–6683. 10.1021/acs.jpclett.1c01401. 34260255 PMC8312498

[ref29] Biasin E.; Nascimento D. R.; Poulter B. I.; Abraham B.; Kunnus K.; Garcia-Esparza A. T.; Nowak S. H.; Kroll T.; Schoenlein R. W.; Alonso-Mori R.; Khalil M.; Govind N.; Sokaras D. Revealing the bonding of solvated Ru complexes with valence-to-core resonant inelastic X-ray scattering. Chem. Sci. 2021, 12, 3713–3725. 10.1039/D0SC06227H. 34163645 PMC8179428

[ref30] Dell’Angela M.; Anniyev T.; Beye M.; Coffee R.; Föhlisch A.; Gladh J.; Katayama T.; Kaya S.; Krupin O.; LaRue J.; Møgelhøj A.; Nordlund D.; Nørskov J. K.; Öberg H.; Ogasawara H.; Öström H.; Pettersson L. G. M.; Schlotter W. F.; Sellberg J. A.; Sorgenfrei F.; Turner J. J.; Wolf M.; Wurth W.; Nilsson A. Real-Time Observation of Surface Bond Breaking with an X-ray Laser. Science 2013, 339, 1302–1305. 10.1126/science.1231711. 23493709

[ref31] Öström H.; Öberg H.; Xin H.; LaRue J.; Beye M.; Dell’Angela M.; Gladh J.; Ng M. L.; Sellberg J. A.; Kaya S.; Mercurio G.; Nordlund D.; Hantschmann M.; Hieke F.; Kühn D.; Schlotter W. F.; Dakovski G. L.; Turner J. J.; Minitti M. P.; Mitra A.; Moeller S. P.; Föhlisch A.; Wolf M.; Wurth W.; Persson M.; Nørskov J. K.; Abild-Pedersen F.; Ogasawara H.; Pettersson L. G. M.; Nilsson A. Probing the transition state region in catalytic CO oxidation on Ru. Science 2015, 347, 978–982. 10.1126/science.1261747. 25722407

[ref32] Jeyachandran Y. L.; Meyer F.; Nagarajan S.; Benkert A.; Bär M.; Blum M.; Yang W.; Reinert F.; Heske C.; Weinhardt L.; Zharnikov M. Ion-Solvation-Induced Molecular Reorganization in Liquid Water Probed by Resonant Inelastic Soft X-ray Scattering. J. Phys. Chem. Lett. 2014, 5, 4143–4148. 10.1021/jz502186a. 26278946

[ref33] Savchenko V.; Brumboiu I. E.; Kimberg V.; Odelius M.; Krasnov P.; Liu J.-C.; Rubensson J.-E.; Björneholm O.; Såthe C.; Gråsjö J.; Dong M.; Pietzsch A.; Föhlisch A.; Schmitt T.; McNally D.; Lu X.; Polyutov S. P.; Norman P.; Iannuzzi M.; Gel’mukhanov F.; Ekholm V. Vibrational resonant inelastic X-ray scattering in liquid acetic acid: a ruler for molecular chain lengths. Sci. Rep. 2021, 11, 4098 10.1038/s41598-021-83248-3. 33602972 PMC7893077

[ref34] Marchenko T.; Carniato S.; Journel L.; Guillemin R.; Kawerk E.; Žitnik M.; Kavčič M.; Bučar K.; Bohinc R.; Petric M.; da Cruz V. V.; Gel’mukhanov F.; Simon M. Electron dynamics in the core-excited CS_2_ molecule revealed through resonant inelastic X-ray scattering spectroscopy. J. Phys.: Conf. Ser. 2015, 635, 112012 10.1088/1742-6596/635/11/112012.

[ref35] Žitnik M.; Kavčič M.; Bohinc R.; Bučar K.; Mihelič A.; Cao W.; Guillemin R.; Journel L.; Marchenko T.; Carniato S.; Kawerk E.; Piancastelli M.; Simon M. Resonant inelastic X-ray spectroscopy of atoms and simple molecules: Satellite features and dependence on energy detuning and photon polarization. J. Electron Spectrosc. Relat. Phenom. 2015, 204, 356–364. 10.1016/j.elspec.2015.05.018.

[ref36] Eckert S.; Vaz da Cruz V.; Ochmann M.; von Ahnen I.; Föhlisch A.; Huse N. Breaking the Symmetry of Pyrimidine: Solvent Effects and Core-Excited State Dynamics. J. Phys. Chem. Lett. 2021, 12, 8637–8643. 10.1021/acs.jpclett.1c01865. 34472857 PMC8436212

[ref37] Gel’mukhanov F.; Ågren H. Resonant inelastic X-ray scattering with symmetry-selective excitation. Phys. Rev. A 1994, 49, 4378–4389. 10.1103/PhysRevA.49.4378. 9910751

[ref38] Skytt P.; Guo J.; Wassdahl N.; Nordgren J.; Luo Y.; Ågren H. Probing symmetry breaking upon core excitation with resonant X-ray fluorescence. Phys. Rev. A 1995, 52, 3572–3576. 10.1103/PhysRevA.52.3572. 9912658

[ref39] Luo Y.; Ågren H.; Gel’mukhanov F.; Guo J.; Skytt P.; Wassdahl N.; Nordgren J. Symmetry-selective resonant inelastic X-ray scattering of C_60_. Phys. Rev. B 1995, 52, 14479–14496. 10.1103/PhysRevB.52.14479. 9980778

[ref40] Glans P.; Gunnelin K.; Skytt P.; Guo J.-H.; Wassdahl N.; Nordgren J.; Ågren H.; Gel’mukhanov F. K.; Warwick T.; Rotenberg E. Resonant X-Ray Emission Spectroscopy of Molecular Oxygen. Phys. Rev. Lett. 1996, 76, 2448–2451. 10.1103/PhysRevLett.76.2448. 10060702

[ref41] Hergenhahn U.; Kugeler O.; Rüdel A.; Rennie E. E.; Bradshaw A. M. Symmetry-Selective Observation of the N 1s Shape Resonance in N_2_. J. Phys. Chem. A 2001, 105, 5704–5708. 10.1021/jp0038456.

[ref42] Kosugi N. Exchange interaction in core excitation of diatomic systems. Chem. Phys. 2003, 289, 117–134. 10.1016/S0301-0104(02)00791-7.

[ref43] Bagus P. S.; Schaefer H. F. Localized and Delocalized 1s Hole States of the O_2_^+^ Molecular Ion. J. Chem. Phys. 2003, 56, 224–226. 10.1063/1.1676850.

[ref44] Ehara M.; Nakatsuji H.; Matsumoto M.; Hatamoto T.; Liu X.-J.; Lischke T.; Prümper G.; Tanaka T.; Makochekanwa C.; Hoshino M.; Tanaka H.; Harries J. R.; Tamenori Y.; Ueda K. Symmetry-dependent vibrational excitation in N 1s photoionization of N_2_: Experiment and theory. J. Chem. Phys. 2006, 124, 124311 10.1063/1.2181144. 16599678

[ref45] Cederbaum L. S.; Domcke W. Localized and delocalized core holes and their interrelation. J. Chem. Phys. 1977, 66, 5084–5086. 10.1063/1.433763.

[ref46] Xu Y.-C.; Wang S.-X.; Du X.-J.; Wang L.-H.; Zhu L.-F. Probing the delocalized core-hole via inner-shell excitation in N_2_. New J. Phys. 2022, 24, 053036 10.1088/1367-2630/ac6bb7.

[ref47] Köppel H.; Domcke W.; Cederbaum L. S. Advances in Chemical Physics; John Wiley & Sons, Ltd, 1984, pp 59–246.

[ref48] Domcke W.; Cederbaum L. Vibronic coupling and symmetry breaking in core electron ionization. Chem. Phys. 1977, 25, 189–196. 10.1016/0301-0104(77)87075-4.

[ref49] Cederbaum L. S. Symmetry breaking and localization in resonant photon emission. J. Chem. Phys. 1995, 103, 562–567. 10.1063/1.470090.

[ref50] Ma Y.; Wassdahl N.; Skytt P.; Guo J.; Nordgren J.; Johnson P. D.; Rubensson J.-E.; Boske T.; Eberhardt W.; Kevan S. D. Soft-X-ray resonant inelastic scattering at the C K edge of diamond. Phys. Rev. Lett. 1992, 69, 2598–2601. 10.1103/PhysRevLett.69.2598. 10046535

[ref51] Skytt P.; Glans P.; Guo J.-H.; Gunnelin K.; Såthe C.; Nordgren J.; Gel’mukhanov F. K.; Cesar A.; Ågren H. Quenching of Symmetry Breaking in Resonant Inelastic X-Ray Scattering by Detuned Excitation. Phys. Rev. Lett. 1996, 77, 5035–5038. 10.1103/PhysRevLett.77.5035. 10062698

[ref52] Cesar A.; Gel’mukhanov F.; Luo Y.; Ågren H.; Skytt P.; Glans P.; Guo J.; Gunnelin K.; Nordgren J. Resonant x-ray scattering beyond the Born–Oppenheimer approximation: Symmetry breaking in the oxygen resonant x-ray emission spectrum of carbon dioxide. J. Chem. Phys. 1997, 106, 3439–3456. 10.1063/1.474111.

[ref53] Gelmukhanov F.; Ågren H. X-ray resonant scattering involving dissociative states. Phys. Rev. A 1996, 54, 379–393. 10.1103/PhysRevA.54.379. 9913488

[ref54] Gel’mukhanov F.; Sałek P.; Privalov T.; Ågren H. Duration of x-ray Raman scattering. Phys. Rev. A 1999, 59, 380–389. 10.1103/PhysRevA.59.380.

[ref55] Björneholm O.; Sundin S.; Svensson S.; Marinho R. R. T.; Naves de Brito A.; Gel’mukhanov F.; Ågren H. Femtosecond Dissociation of Core-Excited HCl Monitored by Frequency Detuning. Phys. Rev. Lett. 1997, 79, 3150–3153. 10.1103/PhysRevLett.79.3150.

[ref56] Sundin S.; Kh. Gel’mukhanov F.; Ågren H.; Osborne S. J.; Kikas A.; Björneholm O.; Ausmees A.; Svensson S. Collapse of Vibrational Structure in the Auger Resonant Raman Spectrum of CO by Frequency Detuning. Phys. Rev. Lett. 1997, 79, 1451–1454. 10.1103/physrevlett.79.1451.

[ref57] Gel’mukhanov F.; Privalov T.; Ågren H. Collapse of vibrational structure in spectra of resonant X-ray Raman scattering. Phys. Rev. A 1997, 56, 256–264. 10.1103/PhysRevA.56.256.

[ref58] Harada Y.; Tokushima T.; Takata Y.; Takeuchi T.; Kitajima Y.; Tanaka S.; Kayanuma Y.; Shin S. Dynamical Symmetry Breaking under Core Excitation in Graphite: Polarization Correlation in Soft X-Ray Recombination Emission. Phys. Rev. Lett. 2004, 93, 017401 10.1103/PhysRevLett.93.017401.

[ref59] Hennies F.; Polyutov S.; Minkov I.; Pietzsch A.; Nagasono M.; Gel’mukhanov F.; Triguero L.; Piancastelli M.-N.; Wurth W.; Ågren H.; Föhlisch A. Nonadiabatic Effects in Resonant Inelastic X-Ray Scattering. Phys. Rev. Lett. 2005, 95, 163002 10.1103/PhysRevLett.95.163002. 16241792

[ref60] Maganas D.; Kristiansen P.; Duda L.-C.; Knop-Gericke A.; DeBeer S.; Schlögl R.; Neese F. Combined Experimental and Ab Initio Multireference Configuration Interaction Study of the Resonant Inelastic X-ray Scattering Spectrum of CO2. J. Phys. Chem. C 2014, 118, 20163–20175. 10.1021/jp505628y.

[ref61] Kramers H. A.; Heisenberg W. Über die Streuung von Strahlung durch Atome. Z. Phys. 1925, 31, 681–708. 10.1007/BF02980624.

[ref62] Dirac P. A. M. The quantum theory of dispersion. Proc. R. Soc. London, Ser. A 1927, 114, 710–728.

[ref63] Tannor D. J.; Heller E. J. Polyatomic Raman scattering for general harmonic potentials. J. Chem. Phys. 1982, 77, 202–218. 10.1063/1.443643.

[ref64] Herzberg G.; Teller E. Schwingungsstruktur der Elektronenübergänge bei mehratomigen Molekülen. Z. Phys. Chem. 1933, 21B, 410–446. 10.1515/zpch-1933-2136.

[ref65] Albrecht A. C. On the Theory of Raman Intensities. J. Chem. Phys. 1961, 34, 1476–1484. 10.1063/1.1701032.

[ref66] Neugebauer J.; Hess B. A. Resonance Raman spectra of uracil based on Kramers–Kronig relations using time-dependent density functional calculations and multireference perturbation theory. J. Chem. Phys. 2004, 120, 11564–11577. 10.1063/1.1697371. 15268191

[ref67] Jensen L.; Autschbach J.; Schatz G. C. Finite lifetime effects on the polarizability within time-dependent density-functional theory. J. Chem. Phys. 2005, 122, 224115 10.1063/1.1929740. 15974659

[ref68] Jensen L.; Zhao L. L.; Autschbach J.; Schatz G. C. Theory and method for calculating resonance Raman scattering from resonance polarizability derivatives. J. Chem. Phys. 2005, 123, 174110 10.1063/1.2046670. 16375520

[ref69] Rappoport D.; Shim S.; Aspuru-Guzik A. Simplified Sum-Over-States Approach for Predicting Resonance Raman Spectra. Application to Nucleic Acid Bases. J. Phys. Chem. Lett. 2011, 2, 1254–1260. 10.1021/jz200413g. 26295418

[ref70] Lee S.; Heller E. J. Time-dependent theory of Raman scattering. J. Chem. Phys. 1979, 71, 4777–4788. 10.1063/1.438316.

[ref71] Thomas M.; Latorre F.; Marquetand P. Resonance Raman spectra of ortho-nitrophenol calculated by real-time time-dependent density functional theory. J. Chem. Phys. 2013, 138, 044101 10.1063/1.4776218. 23387562

[ref72] Mattiat J.; Luber S. Efficient calculation of (resonance) Raman spectra and excitation profiles with real-time propagation. J. Chem. Phys. 2018, 149, 174108 10.1063/1.5051250. 30409007

[ref73] Ovander L. N.; Shadura V. A. Green function method and its application to the resonance Raman effect. J. Raman Spectrosc. 2001, 32, 587–590. 10.1002/jrs.735.

[ref74] Kas J. J.; Rehr J. J.; Soininen J. A.; Glatzel P. Real-space Green’s function approach to resonant inelastic x-ray scattering. Phys. Rev. B 2011, 83, 235114 10.1103/physrevb.83.235114.

[ref75] Guthmuller J.; Champagne B. Resonance Raman Scattering of Rhodamine 6G as Calculated by Time-Dependent Density Functional Theory: Vibronic and Solvent Effects. J. Phys. Chem. A 2008, 112, 3215–3223. 10.1021/jp7112279. 18327928

[ref76] Ma H.; Liu J.; Liang W. Time-Dependent Approach to Resonance Raman Spectra Including Duschinsky Rotation and Herzberg–Teller Effects: Formalism and Its Realistic Applications. J. Chem. Theory Comput. 2012, 8, 4474–4482. 10.1021/ct300640c. 26605607

[ref77] Meyer H.-D.; Manthe U.; Cederbaum L. The multi-configurational time-dependent Hartree approach. Chem. Phys. Lett. 1990, 165, 73–78. 10.1016/0009-2614(90)87014-I.

[ref78] Freibert A.; Mendive-Tapia D.; Huse N.; Vendrell O. Femtosecond x-ray absorption spectroscopy of pyrazine at the nitrogen K-edge: on the validity of the Lorentzian limit. J. Phys. B: At., Mol. Opt. Phys. 2021, 54, 244003 10.1088/1361-6455/ac3846.

[ref79] Cederbaum L. S.; Domcke W.; Schirmer J. Many-body theory of core holes. Phys. Rev. A 1980, 22, 206–222. 10.1103/PhysRevA.22.206.

[ref80] Worth G. A.; Cederbaum L. S. Beyond Born-Oppenheimer: Molecular Dynamics Through a Conical Intersection. Annu. Rev. Phys. Chem. 2004, 55, 127–158. 10.1146/annurev.physchem.55.091602.094335. 15117250

[ref81] Cederbaum L.; Domcke W.; Köppel H.; Von Niessen W. Strong vibronic coupling effects in ionization spectra: The “mystery band” of butatriene. Chem. Phys. 1977, 26, 169–177. 10.1016/0301-0104(77)87041-9.

[ref82] Baer M. Adiabatic and diabatic representations for atom-molecule collisions: Treatment of the collinear arrangement. Chem. Phys. Lett. 1975, 35, 112–118. 10.1016/0009-2614(75)85599-0.

[ref83] Pacher T.; Cederbaum L. S.; Köppel H. Advances in Chemical Physics; John Wiley & Sons, Ltd, 1993, pp 293–391.

[ref84] Purvis G. D.; Bartlett R. J. A full coupled-cluster singles and doubles model: The inclusion of disconnected triples. J. Chem. Phys. 1982, 76, 1910–1918. 10.1063/1.443164.

[ref85] Dunning T. H. Gaussian basis sets for use in correlated molecular calculations. I. The atoms boron through neon and hydrogen. J. Chem. Phys. 1989, 90, 1007–1023. 10.1063/1.456153.

[ref86] Frisch M. J.; Trucks G. W.; Schlegel H. B.; Scuseria G. E.; Robb M. A.; Cheeseman J. R.; Scalmani G.; Barone V.; Petersson G. A.; Nakatsuji H.; Li X.; Caricato M.; Marenich A. V.; Bloino J.; Janesko B. G.; Gomperts R.; Mennucci B.; Hratchian H. P.; Ortiz J. V.; Izmaylov A. F.; Sonnenberg J. L.; Williams-Young D.; Ding F.; Lipparini F.; Egidi F.; Goings J.; Peng B.; Petrone A.; Henderson T.; Ranasinghe D.; Zakrzewski V. G.; Gao J.; Rega N.; Zheng G.; Liang W.; Hada M.; Ehara M.; Toyota K.; Fukuda R.; Hasegawa J.; Ishida M.; Nakajima T.; Honda Y.; Kitao O.; Nakai H.; Vreven T.; Throssell K.; Montgomery J. A.; Peralta J. E.; Ogliaro F.; Bearpark M. J.; Heyd J. J.; Brothers E. N.; Kudin K. N.; Staroverov V. N.; Keith T. A.; Kobayashi R.; Normand J.; Raghavachari K.; Rendell A. P.; Burant J. C.; Iyengar S. S.; Tomasi J.; Cossi M.; Millam J. M.; Klene M.; Adamo C.; Cammi R.; Ochterski J. W.; Martin R. L.; Morokuma K.; Farkas O.; Foresman J. B.; Fox D. J. Gaussian 16. Revision A.03; Gaussian Inc: Wallingford CT, 2016.

[ref87] Epifanovsky E.; Gilbert A. T. B.; Feng X.; Lee J.; Mao Y.; Mardirossian N.; Pokhilko P.; White A. F.; Coons M. P.; Dempwolff A. L.; Gan Z.; Hait D.; Horn P. R.; Jacobson L. D.; Kaliman I.; Kussmann J.; Lange A. W.; Lao K. U.; Levine D. S.; Liu J.; McKenzie S. C.; Morrison A. F.; Nanda K. D.; Plasser F.; Rehn D. R.; Vidal M. L.; You Z.-Q.; Zhu Y.; Alam B.; Albrecht B. J.; Aldossary A.; Alguire E.; Andersen J. H.; Athavale V.; Barton D.; Begam K.; Behn A.; Bellonzi N.; Bernard Y. A.; Berquist E. J.; Burton H. G. A.; Carreras A.; Carter-Fenk K.; Chakraborty R.; Chien A. D.; Closser K. D.; Cofer-Shabica V.; Dasgupta S.; de Wergifosse M.; Deng J.; Diedenhofen M.; Do H.; Ehlert S.; Fang P.-T.; Fatehi S.; Feng Q.; Friedhoff T.; Gayvert J.; Ge Q.; Gidofalvi G.; Goldey M.; Gomes J.; González-Espinoza C. E.; Gulania S.; Gunina A. O.; Hanson-Heine M. W. D.; Harbach P. H. P.; Hauser A.; Herbst M. F.; Hernández Vera M.; Hodecker M.; Holden Z. C.; Houck S.; Huang X.; Hui K.; Huynh B. C.; Ivanov M.; Jász Á.; Ji H.; Jiang H.; Kaduk B.; Kähler S.; Khistyaev K.; Kim J.; Kis G.; Klunzinger P.; Koczor-Benda Z.; Koh J. H.; Kosenkov D.; Koulias L.; Kowalczyk T.; Krauter C. M.; Kue K.; Kunitsa A.; Kus T.; Ladjánszki I.; Landau A.; Lawler K. V.; Lefrancois D.; Lehtola S.; Li R. R.; Li Y.-P.; Liang J.; Liebenthal M.; Lin H.-H.; Lin Y.-S.; Liu F.; Liu K.-Y.; Loipersberger M.; Luenser A.; Manjanath A.; Manohar P.; Mansoor E.; Manzer S. F.; Mao S.-P.; Marenich A. V.; Markovich T.; Mason S.; Maurer S. A.; McLaughlin P. F.; Menger M. F. S. J.; Mewes J.-M.; Mewes S. A.; Morgante P.; Mullinax J. W.; Oosterbaan K. J.; Paran G.; Paul A. C.; Paul S. K.; Pavošević F.; Pei Z.; Prager S.; Proynov E. I.; Rák Á.; Ramos-Cordoba E.; Rana B.; Rask A. E.; Rettig A.; Richard R. M.; Rob F.; Rossomme E.; Scheele T.; Scheurer M.; Schneider M.; Sergueev N.; Sharada S. M.; Skomorowski W.; Small D. W.; Stein C. J.; Su Y.-C.; Sundstrom E. J.; Tao Z.; Thirman J.; Tornai G. J.; Tsuchimochi T.; Tubman N. M.; Veccham S. P.; Vydrov O.; Wenzel J.; Witte J.; Yamada A.; Yao K.; Yeganeh S.; Yost S. R.; Zech A.; Zhang I. Y.; Zhang X.; Zhang Y.; Zuev D.; Aspuru-Guzik A.; Bell A. T.; Besley N. A.; Bravaya K. B.; Brooks B. R.; Casanova D.; Chai J.-D.; Coriani S.; Cramer C. J.; Cserey G.; DePrince A. E.; DiStasio R. A.; Dreuw A.; Dunietz B. D.; Furlani T. R.; Goddard W. A.; Hammes-Schiffer S.; Head-Gordon T.; Hehre W. J.; Hsu C. P.; Jagau T. C.; Jung Y.; Klamt A.; Kong J.; Lambrecht D. S.; Liang W.; Mayhall N. J.; McCurdy C. W.; Neaton J. B.; Ochsenfeld C.; Parkhill J. A.; Peverati R.; Rassolov V. A.; Shao Y.; Slipchenko L. V.; Stauch T.; Steele R. P.; Subotnik J. E.; Thom A. J. W.; Tkatchenko A.; Truhlar D. G.; Van Voorhis T.; Wesolowski T. A.; Whaley K. B.; Woodcock H. L.; Zimmerman P. M.; Faraji S.; Gill P. M. W.; Head-Gordon M.; Herbert J. M.; Krylov A. I. Software for the frontiers of quantum chemistry: An overview of developments in the Q-Chem 5 package. J. Chem. Phys. 2021, 155, 084801 10.1063/5.0055522. 34470363 PMC9984241

[ref88] Stanton J. F.; Bartlett R. J. The equation of motion coupled-cluster method. A systematic biorthogonal approach to molecular excitation energies, transition probabilities, and excited state properties. J. Chem. Phys. 1993, 98, 7029–7039. 10.1063/1.464746.

[ref89] Vidal M.; Feng X.; Epifanovsky E.; Krylov A. I.; Coriani S. New and Efficient Equation-of-Motion Coupled-Cluster Framework for Core-Excited and Core-Ionized States. J. Chem. Theory Comput. 2019, 15, 3117–3133. 10.1021/acs.jctc.9b00039. 30964297

[ref90] Worth G. A.; Beck M. H.; Jäckle A.; Meyer H.; Otto F.; Brill M.; Vendrell O. The MCTDH Package. version 8.6, 2020. http://mctdh.uni-hd.de. (accessed date 21.01.2024).

[ref91] Williams S. O.; Imre D. G. Raman spectroscopy: time-dependent pictures. J. Phys. Chem. 1988, 92, 3363–3374. 10.1021/j100323a012.

[ref92] Tannor D. J. Introduction to Quantum Mechanics: A Time-Dependent Perspective; University Science Books; 1st ed., 2007.

[ref93] Vendrell O.; Meyer H.-D. Multilayer multiconfiguration time-dependent Hartree method: Implementation and applications to a Henon–Heiles Hamiltonian and to pyrazine. J. Chem. Phys. 2011, 134, 044135 10.1063/1.3535541. 21280715

[ref94] Wang H.; Thoss M. Multilayer formulation of the multiconfiguration time-dependent Hartree theory. J. Chem. Phys. 2003, 119, 1289–1299. 10.1063/1.1580111.

[ref95] Manthe U. A multilayer multiconfigurational time-dependent Hartree approach for quantum dynamics on general potential energy surfaces. J. Chem. Phys. 2008, 128, 164116 10.1063/1.2902982. 18447430

[ref96] Meyer H.-D. Studying molecular quantum dynamics with the multiconfiguration time-dependent Hartree method. Wiley Interdiscip. Rev.: Comput. Mol. Sci. 2012, 2, 351–374. 10.1002/wcms.87.

[ref97] Dirac P. A. M. Note on Exchange Phenomena in the Thomas Atom. Math. Proc. Cambridge Philos. Soc. 1930, 26, 376–385. 10.1017/S0305004100016108.

[ref98] Frenkel J.. In Wave mechanics; advanced general theory; The international series of monographs on physics; The Clarendon press: Oxford, 1934.

[ref99] Innes K.; Ross I.; Moomaw W. R. Electronic states of azabenzenes and azanaphthalenes: A revised and extended critical review. J. Mol. Spectrosc. 1988, 132, 492–544. 10.1016/0022-2852(88)90343-8.

[ref100] Wilson E. B. The Normal Modes and Frequencies of Vibration of the Regular Plane Hexagon Model of the Benzene Molecule. Phys. Rev. 1934, 45, 706–714. 10.1103/PhysRev.45.706.

[ref101] Prince K. C.; Vondráček M.; Karvonen J.; Coreno M.; Camilloni R.; Avaldi L.; de Simone M. A critical comparison of selected 1s and 2p core hole widths. J. Electron Spectrosc. Relat. Phenom. 1999, 101–103, 141–147. 10.1016/S0368-2048(98)00436-8.

[ref102] Sala M.; Lasorne B.; Gatti F.; Guérin S. The role of the low-lying dark nπ* states in the photophysics of pyrazine: a quantum dynamics study. Phys. Chem. Chem. Phys. 2014, 16, 15957–15967. 10.1039/C4CP02165G. 24964033

[ref103] Tsuru S.; Vidal M. L.; Pápai M.; Krylov A. I.; Møller K. B.; Coriani S. Time-resolved near-edge X-ray absorption fine structure of pyrazine from electronic structure and nuclear wave packet dynamics simulations. J. Chem. Phys. 2019, 151, 124114 10.1063/1.5115154. 31575192

[ref104] Heller E. J. Quantum corrections to classical photodissociation models. J. Chem. Phys. 1978, 68, 2066–2075. 10.1063/1.436029.

[ref105] Schinke R. Photodissociation Dynamics: Spectroscopy and Fragmentation of Small Polyatomic Molecules. In Cambridge Monographs on Atomic, Molecular and Chemical Physics; Cambridge University Press, 1993.

[ref106] Heller E. J.; Sundberg R.; Tannor D. Simple aspects of Raman scattering. J. Phys. Chem. 1982, 86, 1822–1833. 10.1021/j100207a018.

