# Supplementary material for: Time-Dependent Resonant Inelastic X-ray Scattering of Pyrazine at the Nitrogen K-Edge: A Quantum Dynamics Approach
Source: J Chem Theory Comput. 2024 Feb 5;20(5):2167–80. doi: 10.1021/acs.jctc.3c01259 (PMC10938531; doi:10.1021/acs.jctc.3c01259)
Supplement: Supplementary file 1 — ct3c01259_si_001.pdf [file ct3c01259_si_001.pdf]

Supplementary Information: Time-dependent Resonant Inelastic X-ray Scattering of Pyrazine at the Nitrogen K-edge: A Quantum Dynamics Approach

Antonia Freibert,[*,†,‡] David Mendive-Tapia,[‡] Nils Huse,[†] and Oriol Vendrell[*,‡]

†*Department of Physics, University of Hamburg, Luruper Chaussee 149, 22761 Hamburg, Germany*

‡*Theoretical Chemistry, Institute of Physical Chemistry, Heidelberg University, Im Neuenheimer Feld 229, 69120, Heidelberg*

E-mail: afreiber@physnet.uni-hamburg.de; oriol.vendrell@uni-heidelberg.de

A full list of on-diagonal linear intrastate coupling constants $\kappa_i^{(n)}$, off-diagonal linear interstate coupling constants $\lambda_i^{(nm)}$ as well as on-diagonal bilinear (quadratic) coupling constants $\gamma_i^{(n)}$ and the parameters concerning the Morse potential are provided in the form of a MCTDH operator file. Details on how to read this file can be found in the MCTDH documentation.

```
OP_DEFINE-SECTION
  TITLE
    24D Pyrazine 22States
  END-TITLE
END-OP_DEFINE-SECTION

PARAMETER-SECTION

#lifetime
#corresponds approx to 0.11ev line width (see Prince et al)
L_t = 8, fs
L = 2*L_t
#exponential d
L_e = 1/L

#frequencies

omega_1                =   0.04345 , ev
omega_2                =   0.05276 , ev
omega_3                =   0.07494 , ev
omega_4                =   0.08789 , ev
omega_5                =   0.09138 , ev
omega_6                =   0.09984 , ev
```

```
omega_7                 =   0.11696 , ev

omega_8                 =   0.11777 , ev

omega_9                 =   0.12153 , ev

omega_10                =   0.12804 , ev

omega_11                =   0.12868 , ev

omega_12                =   0.13525 , ev

omega_13                =   0.14203 , ev

omega_14                =   0.14407 , ev

omega_15                =   0.15542 , ev

omega_16                =   0.16945 , ev

omega_17                =   0.17867 , ev

omega_18                =   0.18815 , ev

omega_19                =   0.19777 , ev

omega_20                =   0.20453 , ev

omega_21                =   0.39627 , ev

omega_22                =   0.39634 , ev

omega_23                =   0.39840 , ev

omega_24                =   0.39899 , ev

#energies


E1                      =   0.00000 , ev

E2                      =   4.32244 , ev

E3                      =   5.06570 , ev

E4                      =   5.12950 , ev

E5                      =   6.00695 , ev

E6                      =   6.68251 , ev
```

```
E7                       =   6.90526 , ev

E8                       =   7.02394 , ev

E9                       =   7.10984 , ev

E10                      =   7.28000 , ev

E11                      =   7.47034 , ev

E12                      =   7.66268 , ev

E13                      =   7.94469 , ev

E14                      =   8.00322 , ev

E15                      =   8.03587 , ev

E16                      =   8.12045 , ev

E17                      =   8.13950 , ev

E18                      =   8.35338 , ev

E19                      =   8.35554 , ev

E20                      =   8.53117 , ev


X1                       = 402.30130 , ev

X2                       = 402.30260 , ev


#on-diagonal linear coupling constants (kappa)


kappa2_3                 =   0.09079 , ev

kappa2_11                =  -0.07481 , ev

kappa2_15                =   0.12181 , ev

kappa2_20                =  -0.13183 , ev

kappa3_3                 =  -0.11182 , ev

kappa3_11                =  -0.21403 , ev
```

```
kappa3_15              =  -0.00427 , ev
kappa3_20              =  -0.02891 , ev
kappa4_3               =   0.17725 , ev
kappa4_11              =  -0.13295 , ev
kappa4_15              =  -0.00671 , ev
kappa4_20              =  -0.37844 , ev
kappa5_3               =   0.20914 , ev
kappa5_11              =  -0.14007 , ev
kappa5_15              =   0.15338 , ev
kappa5_20              =   0.04179 , ev

#analytical EOM-CCSD/aug-cc-pvdz values at FC
kappa6_3               =  -0.03881 , ev
kappa6_11              =  -0.16883 , ev
kappa6_15              =  -0.05117 , ev
kappa6_20              =  -0.17885 , ev
kappa6_24              =   0.03733 , ev
kappa7_3               =  -0.08009 , ev
kappa7_11              =  -0.06635 , ev
kappa7_15              =  -0.05980 , ev
kappa7_20              =  -0.15619 , ev
kappa7_24              =  -0.00580 , ev
kappa8_3               =   0.28951 , ev
kappa8_11              =  -0.15753 , ev
kappa8_15              =  -0.01717 , ev
kappa8_20              =  -0.16253 , ev
kappa8_24              =   0.07464 , ev
```

```
kappa9_3            =  -0.07984 , ev
kappa9_11           =  -0.06642 , ev
kappa9_15           =  -0.05977 , ev
kappa9_20           =  -0.15632 , ev
kappa9_24           =  -0.00584 , ev
kappa10_3           =   0.17197 , ev
kappa10_11          =   0.03820 , ev
kappa10_15          =   0.03730 , ev
kappa10_20          =  -0.25809 , ev
kappa10_24          =  -0.00149 , ev
kappa11_3           =   0.16987 , ev
kappa11_11          =   0.00664 , ev
kappa11_15          =  -0.02059 , ev
kappa11_20          =  -0.21801 , ev
kappa11_24          =   0.01102 , ev
kappa12_3           =  -0.06130 , ev
kappa12_11          =  -0.07675 , ev
kappa12_15          =  -0.02988 , ev
kappa12_20          =  -0.17033 , ev
kappa12_24          =  -0.01339 , ev
kappa13_3           =   0.01687 , ev
kappa13_11          =  -0.17105 , ev
kappa13_15          =   0.01355 , ev
kappa13_20          =  -0.04513 , ev
kappa13_24          =   0.03582 , ev
kappa14_3           =   0.17506 , ev
kappa14_11          =   0.01019 , ev
```

```
kappa14_15          =  -0.00477 , ev
kappa14_20          =  -0.23923 , ev
kappa14_24          =  -0.00292 , ev
kappa15_3           =   0.16932 , ev
kappa15_11          =   0.01919 , ev
kappa15_15          =   0.07767 , ev
kappa15_20          =  -0.26638 , ev
kappa15_24          =  -0.00749 , ev
kappa16_3           =  -0.05003 , ev
kappa16_11          =  -0.08945 , ev
kappa16_15          =  -0.09501 , ev
kappa16_20          =  -0.16226 , ev
kappa16_24          =  -0.00674 , ev
kappa17_3           =   0.03746 , ev
kappa17_11          =  -0.16458 , ev
kappa17_15          =   0.12776 , ev
kappa17_20          =   0.14672 , ev
kappa17_24          =   0.00147 , ev
kappa18_3           =  -0.06741 , ev
kappa18_11          =  -0.08585 , ev
kappa18_15          =   0.00540 , ev
kappa18_20          =  -0.18250 , ev
kappa18_24          =  -0.02424 , ev
kappa19_3           =  -0.20211 , ev
kappa19_11          =  -0.06997 , ev
kappa19_15          =   0.03820 , ev
kappa19_20          =   0.50935 , ev
```

```
kappa19_24            =  -0.21177 , ev
kappa20_3             =   0.20212 , ev
kappa20_11            =  -0.06999 , ev
kappa20_15            =   0.03829 , ev
kappa20_20            =   0.50939 , ev
kappa20_24            =  -0.21175 , ev

#fittet kappa core-hole states
kappax1_3              =   0.02738 , ev
kappax1_11             =  -0.04034 , ev
kappax1_15             =   0.05568 , ev
kappax1_20             =   0.10433 , ev
kappax2_3              =   0.02627 , ev
kappax2_11             =  -0.04050 , ev
kappax2_15             =   0.05619 , ev
kappax2_20             =   0.10457 , ev

#off-diagonal linear coupling constants (lambda)

lambda2_3_7           =   0.17641 , ev
lambda2_4_16          =  -0.06628 , ev
lambda2_4_19          =  -0.20934 , ev
lambda2_4_21          =   0.01410 , ev
lambda2_5_10          =  -0.18110 , ev
lambda2_5_14          =   0.09724 , ev
lambda2_5_18          =   0.10545 , ev
lambda2_5_22          =   0.00120 , ev
```

```
lambda3_4_5          = -0.05970 , ev
lambda3_4_8          =  0.00252 , ev
lambda3_5_9          = -0.04874 , ev
lambda4_5_12         =  0.06181 , ev
lambda4_5_13         = -0.09166 , ev
lambda4_5_17         =  0.07303 , ev

#analytical EOM-CCSD/aug-cc-pvdz NACs at FC

lambda6_7_10         =  0.01791 , ev
lambda6_7_14         =  0.00458 , ev
lambda6_7_18         =  0.00514 , ev
lambda6_7_22         = -0.00267 , ev
lambda6_8_7          =  0.00820 , ev
lambda6_9_7          =  0.26807 , ev
lambda6_10_12        = -0.00351 , ev
lambda6_10_13        =  0.04370 , ev
lambda6_10_17        =  0.00913 , ev
lambda6_10_23        =  0.01145 , ev
lambda6_11_10        =  0.07891 , ev
lambda6_11_14        = -0.00457 , ev
lambda6_11_18        =  0.00599 , ev
lambda6_11_22        = -0.00161 , ev
lambda6_12_2         =  0.01028 , ev
lambda6_12_6         = -0.00455 , ev
lambda6_13_12        = -0.00227 , ev
lambda6_13_13        =  0.00267 , ev
```

```
lambda6_13_17          =   0.00093 , ev
lambda6_13_23          =   0.00854 , ev
lambda6_14_4           =   0.00403 , ev
lambda6_14_16          =  -0.00117 , ev
lambda6_14_19          =   0.00919 , ev
lambda6_14_21          =  -0.01303 , ev
lambda6_15_3           =  -0.00900 , ev
lambda6_15_11          =   0.00905 , ev
lambda6_15_15          =  -0.00265 , ev
lambda6_15_20          =   0.01136 , ev
lambda6_15_24          =   0.00552 , ev
lambda6_16_1           =  -0.01143 , ev
lambda6_16_9           =  -0.00412 , ev
lambda6_17_10          =  -0.02671 , ev
lambda6_17_14          =   0.00180 , ev
lambda6_17_18          =  -0.00274 , ev
lambda6_17_22          =   0.00137 , ev
lambda6_18_7           =   0.01352 , ev
lambda6_19_1           =  -0.00311 , ev
lambda6_19_9           =  -0.00728 , ev
lambda6_20_5           =   0.12575 , ev
lambda6_20_8           =  -0.01783 , ev

lambda7_8_1            =  -0.06358 , ev
lambda7_8_9            =  -0.00856 , ev
lambda7_9_1            =  -0.08216 , ev
lambda7_9_9            =   0.03833 , ev
```

```
lambda7_10_4          =  -0.00547 , ev
lambda7_10_16         =   0.00557 , ev
lambda7_10_19         =   0.01282 , ev
lambda7_10_21         =   0.00568 , ev
lambda7_11_3          =   0.03910 , ev
lambda7_11_11         =   0.03576 , ev
lambda7_11_15         =   0.03244 , ev
lambda7_11_20         =   0.05961 , ev
lambda7_11_24         =  -0.00620 , ev
lambda7_12_5          =   0.01707 , ev
lambda7_12_8          =  -0.01678 , ev
lambda7_13_4          =   0.04642 , ev
lambda7_13_16         =   0.04537 , ev
lambda7_13_19         =   0.19671 , ev
lambda7_13_21         =   0.00557 , ev
lambda7_14_12         =   0.00999 , ev
lambda7_14_13         =   0.00048 , ev
lambda7_14_17         =  -0.01120 , ev
lambda7_14_23         =   0.00300 , ev
lambda7_15_10         =   0.00999 , ev
lambda7_15_14         =  -0.00161 , ev
lambda7_15_18         =   0.00337 , ev
lambda7_15_22         =  -0.00118 , ev
lambda7_16_7          =  -0.03726 , ev
lambda7_17_3          =   0.01057 , ev
lambda7_17_11         =   0.00903 , ev
lambda7_17_15         =   0.11446 , ev
```

```
lambda7_17_20           =   0.23332 , ev
lambda7_17_24           =  -0.00416 , ev
lambda7_18_1            =  -0.16561 , ev
lambda7_18_9            =   0.07083 , ev
lambda7_19_7            =   0.01285 , ev
lambda7_20_2            =  -0.01542 , ev
lambda7_20_6            =  -0.01165 , ev

lambda8_9_3             =  -0.00993 , ev
lambda8_9_11            =   0.00255 , ev
lambda8_9_15            =  -0.00097 , ev
lambda8_9_20            =   0.00156 , ev
lambda8_9_24            =  -0.00053 , ev
lambda8_10_2            =   0.00158 , ev
lambda8_10_6            =  -0.00311 , ev
lambda8_11_1            =   0.03954 , ev
lambda8_11_9            =  -0.00680 , ev
lambda8_12_12           =   0.00046 , ev
lambda8_12_13           =  -0.00109 , ev
lambda8_12_17           =   0.00039 , ev
lambda8_12_23           =  -0.00247 , ev
lambda8_13_2            =   0.18572 , ev
lambda8_13_6            =   0.04772 , ev
lambda8_14_5            =   0.00036 , ev
lambda8_14_8            =  -0.00215 , ev
lambda8_15_7            =  -0.00886 , ev
lambda8_16_10           =  -0.00111 , ev
```

```
lambda8_16_14          =   0.00038 , ev
lambda8_16_18          =  -0.00003 , ev
lambda8_16_22          =  -0.00343 , ev
lambda8_17_1           =  -0.08957 , ev
lambda8_17_9           =  -0.03919 , ev
lambda8_18_3           =  -0.01044 , ev
lambda8_18_11          =   0.00160 , ev
lambda8_18_15          =   0.00045 , ev
lambda8_18_20          =  -0.00003 , ev
lambda8_18_24          =  -0.00396 , ev
lambda8_19_10          =   0.00770 , ev
lambda8_19_14          =  -0.00598 , ev
lambda8_19_18          =  -0.01030 , ev
lambda8_19_22          =   0.00654 , ev
lambda8_20_4           =   0.00120 , ev
lambda8_20_16          =   0.00046 , ev
lambda8_20_19          =  -0.00165 , ev
lambda8_20_21          =   0.00413 , ev

lambda9_10_2           =   0.00918 , ev
lambda9_10_6           =  -0.00609 , ev
lambda9_11_1           =   0.04217 , ev
lambda9_11_9           =  -0.00244 , ev
lambda9_12_12          =  -0.00621 , ev
lambda9_12_13          =  -0.04028 , ev
lambda9_12_16          =   0.01675 , ev
lambda9_12_23          =   0.00694 , ev
```

```
lambda9_13_2      =  0.02905 , ev
lambda9_13_6      = -0.00057 , ev
lambda9_14_5      =  0.01387 , ev
lambda9_14_8      = -0.00780 , ev
lambda9_15_7      = -0.00231 , ev
lambda9_16_10     = -0.00334 , ev
lambda9_16_14     =  0.00640 , ev
lambda9_16_18     = -0.01897 , ev
lambda9_16_22     = -0.00208 , ev
lambda9_17_1      =  0.03365 , ev
lambda9_17_9      = -0.02079 , ev
lambda9_18_3      = -0.01112 , ev
lambda9_18_11     =  0.01167 , ev
lambda9_18_15     = -0.01324 , ev
lambda9_18_20     =  0.01188 , ev
lambda9_18_24     =  0.01369 , ev
lambda9_19_10     =  0.00270 , ev
lambda9_19_14     =  0.00021 , ev
lambda9_19_18     = -0.00018 , ev
lambda9_19_22     = -0.00667 , ev
lambda9_20_4      = -0.00119 , ev
lambda9_20_16     =  0.02113 , ev
lambda9_20_19     =  0.01637 , ev
lambda9_20_21     =  0.01937 , ev

lambda10_11_4     =  0.01113 , ev
lambda10_11_16    =  0.00083 , ev
```

```
lambda10_11_19         =   0.00554 , ev
lambda10_11_21         =   0.01978 , ev
lambda10_12_7          =   0.26330 , ev
lambda10_13_3          =   0.00288 , ev
lambda10_13_11         =  -0.00184 , ev
lambda10_13_15         =   0.00320 , ev
lambda10_13_20         =   0.00256 , ev
lambda10_13_24         =  -0.00844 , ev
lambda10_14_10         =   0.07071 , ev
lambda10_14_14         =  -0.06674 , ev
lambda10_14_18         =   0.01218 , ev
lambda10_14_22         =  -0.02982 , ev
lambda10_15_12         =   0.00128 , ev
lambda10_15_13         =  -0.04956 , ev
lambda10_15_17         =  -0.02883 , ev
lambda10_15_23         =  -0.02254 , ev
lambda10_16_5          =   0.00049 , ev
lambda10_16_8          =   0.00441 , ev
lambda10_17_4          =  -0.02291 , ev
lambda10_17_16         =   0.01410 , ev
lambda10_17_17         =  -0.00385 , ev
lambda10_17_21         =  -0.02066 , ev
lambda10_18_2          =  -0.00655 , ev
lambda10_18_6          =   0.00088 , ev
lambda10_19_5          =   0.00052 , ev
lambda10_19_8          =  -0.00018 , ev
lambda10_20_1          =  -0.01219 , ev
```

```
lambda10_20_9          =  -0.00545 , ev


lambda11_12_5          =   0.00531 , ev
lambda11_12_8          =   0.00133 , ev
lambda11_13_4          =  -0.00841 , ev
lambda11_13_16         =   0.00250 , ev
lambda11_13_19         =   0.00864 , ev
lambda11_13_21         =   0.00904 , ev
lambda11_14_12         =   0.06123 , ev
lambda11_14_13         =   0.04514 , ev
lambda11_14_17         =  -0.00681 , ev
lambda11_14_23         =   0.01442 , ev
lambda11_15_10         =   0.01818 , ev
lambda11_15_14         =  -0.01690 , ev
lambda11_15_18         =   0.01985 , ev
lambda11_15_22         =   0.00864 , ev
lambda11_16_7          =  -0.27856 , ev
lambda11_17_3          =  -0.04791 , ev
lambda11_17_11         =  -0.04251 , ev
lambda11_17_15         =   0.03049 , ev
lambda11_17_20         =   0.05795 , ev
lambda11_17_24         =   0.01486 , ev
lambda11_18_1          =   0.05233 , ev
lambda11_18_9          =  -0.01557 , ev
lambda11_19_7          =   0.00152 , ev
lambda11_20_2          =  -0.01336 , ev
lambda11_20_6          =   0.00766 , ev
```

```
lambda12_13_7          =   0.01050 , ev
lambda12_14_1          =   0.01284 , ev
lambda12_14_9          =   0.00580 , ev
lambda12_15_2          =  -0.00773 , ev
lambda12_15_6          =   0.00224 , ev
lambda12_16_4          =  -0.02628 , ev
lambda12_16_16         =   0.02792 , ev
lambda12_16_19         =   0.01837 , ev
lambda12_16_21         =  -0.02587 , ev
lambda12_17_5          =  -0.00726 , ev
lambda12_17_8          =   0.01230 , ev
lambda12_18_12         =   0.00451 , ev
lambda12_18_13         =   0.00867 , ev
lambda12_18_17         =  -0.03727 , ev
lambda12_18_23         =  -0.02668 , ev
lambda12_19_4          =  -0.00343 , ev
lambda12_19_16         =   0.00082 , ev
lambda12_19_19         =  -0.00157 , ev
lambda12_19_21         =   0.00589 , ev
lambda12_20_10         =  -0.02221 , ev
lambda12_20_14         =   0.08441 , ev
lambda12_20_18         =  -0.01223 , ev
lambda12_20_22         =   0.03253 , ev

lambda13_14_10         =  -0.00384 , ev
lambda13_14_14         =   0.00320 , ev
```

```
lambda13_14_18          =   0.00317 , ev
lambda13_14_22          =  -0.00557 , ev
lambda13_15_12          =   0.00579 , ev
lambda13_15_13          =  -0.02074 , ev
lambda13_15_17          =  -0.00413 , ev
lambda13_15_23          =  -0.01118 , ev
lambda13_16_5           =  -0.01208 , ev
lambda13_16_8           =   0.03224 , ev
lambda13_17_4           =  -0.01407 , ev
lambda13_17_16          =   0.03959 , ev
lambda13_17_19          =   0.12534 , ev
lambda13_17_21          =   0.01891 , ev
lambda13_18_2           =   0.02712 , ev
lambda13_18_6           =   0.01345 , ev
lambda13_19_5           =  -0.06891 , ev
lambda13_19_8           =   0.05862 , ev
lambda13_20_1           =  -0.11025 , ev
lambda13_20_9           =  -0.04001 , ev

lambda14_15_4           =   0.00281 , ev
lambda14_15_16          =   0.00630 , ev
lambda14_15_19          =   0.00596 , ev
lambda14_15_21          =  -0.03506 , ev
lambda14_16_2           =  -0.00882 , ev
lambda14_16_6           =   0.00525 , ev
lambda14_17_12          =   0.00091 , ev
lambda14_17_13          =  -0.00598 , ev
```

```
lambda14_17_17          =   0.00754 , ev
lambda14_17_23          =  -0.01842 , ev
lambda14_18_5           =  -0.00386 , ev
lambda14_18_8           =  -0.00001 , ev
lambda14_19_2           =  -0.00105 , ev
lambda14_19_6           =  -0.00019 , ev
lambda14_20_7           =  -0.26988 , ev

lambda15_16_1           =  -0.00466 , ev
lambda15_16_9           =   0.00060 , ev
lambda15_17_10          =   0.03312 , ev
lambda15_17_14          =  -0.01250 , ev
lambda15_17_18          =   0.01773 , ev
lambda15_17_22          =  -0.01891 , ev
lambda15_18_7           =   0.25171 , ev
lambda15_19_1           =   0.01676 , ev
lambda15_19_9           =   0.01311 , ev
lambda15_20_5           =  -0.00020 , ev
lambda15_20_8           =  -0.00157 , ev

lambda16_17_7           =   0.03172 , ev
lambda16_18_10          =   0.00054 , ev
lambda16_18_14          =   0.00310 , ev
lambda16_18_18          =  -0.00248 , ev
lambda16_18_22          =  -0.02806 , ev
lambda16_19_3           =   0.00798 , ev
lambda16_19_11          =  -0.00099 , ev
```

```
lambda16_19_15          =  -0.00830 , ev
lambda16_19_20          =  -0.04292 , ev
lambda16_19_24          =   0.01901 , ev
lambda16_20_12          =   0.05986 , ev
lambda16_20_13          =  -0.02096 , ev
lambda16_20_17          =   0.00174 , ev
lambda16_20_23          =   0.01210 , ev

lambda17_18_1           =   0.09621 , ev
lambda17_18_9           =  -0.01870 , ev
lambda17_19_7           =   0.13390 , ev
lambda17_20_2           =   0.04830 , ev
lambda17_20_6           =   0.00848 , ev

lambda18_19_10          =   0.00742 , ev
lambda18_19_14          =   0.00300 , ev
lambda18_19_17          =   0.00882 , ev
lambda18_19_22          =   0.00955 , ev
lambda18_20_4           =   0.00767 , ev
lambda18_20_16          =  -0.01765 , ev
lambda18_20_19          =  -0.01057 , ev
lambda18_20_21          =   0.04863 , ev

lambda19_20_12          =   0.00056 , ev
lambda19_20_13          =  -0.00345 , ev
lambda19_20_17          =   0.00137 , ev
lambda19_20_23          =  -0.00927 , ev
```

```
#fittet NACs for core-hole states

lambdax1_x2_10           =   0.08680 , ev
lambdax1_x2_14           =   0.01326 , ev
lambdax1_x2_18           =   0.09910 , ev
lambdax1_x2_22           =   0.03014 , ev


#on-diagonal bilinear coupling constants (gamma)

gamma2_1_1             =   0.03452 , ev
gamma2_2_2             =  -0.01893 , ev
gamma2_3_3             =   0.00267 , ev
gamma2_4_4             =  -0.01476 , ev
gamma2_5_5             =  -0.04539 , ev
gamma2_6_6             =   0.02674 , ev
gamma2_7_7             =   0.00120 , ev
gamma2_8_8             =   0.00443 , ev
gamma2_9_9             =  -0.00679 , ev
gamma2_10_10           =  -0.02824 , ev
gamma2_11_11           =   0.00242 , ev
gamma2_12_12           =   0.00471 , ev
gamma2_13_13           =   0.07069 , ev
gamma2_14_14           =  -0.00749 , ev
gamma2_15_15           =  -0.00230 , ev
gamma2_16_16           =  -0.00628 , ev
```

```
gamma2_17_17          =  -0.01667 , ev
gamma2_18_18          =  -0.00781 , ev
gamma2_19_19          =  -0.02333 , ev
gamma2_20_20          =   0.00228 , ev
gamma2_21_21          =   0.05903 , ev
gamma2_22_22          =   0.06024 , ev
gamma2_23_23          =   0.06098 , ev
gamma3_1_1            =  -0.02903 , ev
gamma3_2_2            =  -0.05323 , ev
gamma3_3_3            =  -0.01714 , ev
gamma3_4_4            =  -0.00921 , ev
gamma3_5_5            =  -0.05246 , ev
gamma3_6_6            =   0.01932 , ev
gamma3_7_7            =   0.00131 , ev
gamma3_8_8            =  -0.00758 , ev
gamma3_9_9            =   0.00639 , ev
gamma3_10_10          =  -0.00701 , ev
gamma3_11_11          =   0.00674 , ev
gamma3_12_12          =   0.01054 , ev
gamma3_13_13          =   0.10222 , ev
gamma3_14_14          =  -0.00693 , ev
gamma3_15_15          =  -0.01910 , ev
gamma3_16_16          =   0.00516 , ev
gamma3_17_17          =   0.00347 , ev
gamma3_18_18          =  -0.00182 , ev
gamma3_19_19          =   0.00395 , ev
gamma3_20_20          =   0.01406 , ev
```

```
gamma3_21_21            =   0.06107 , ev
gamma3_22_22            =   0.06123 , ev
gamma3_23_23            =   0.06017 , ev
gamma4_1_1              =  -0.02195 , ev
gamma4_2_2              =   0.02623 , ev
gamma4_3_3              =   0.01929 , ev
gamma4_4_4              =  -0.03102 , ev
gamma4_5_5              =  -0.05246 , ev
gamma4_6_6              =   0.00409 , ev
gamma4_7_7              =  -0.05024 , ev
gamma4_8_8              =  -0.02844 , ev
gamma4_9_9              =  -0.04634 , ev
gamma4_10_10            =  -0.05358 , ev
gamma4_11_11            =   0.00583 , ev
gamma4_12_12            =   0.00436 , ev
gamma4_13_13            =   0.08289 , ev
gamma4_14_14            =  -0.01173 , ev
gamma4_15_15            =   0.02741 , ev
gamma4_16_16            =  -0.00631 , ev
gamma4_17_17            =  -0.00963 , ev
gamma4_18_18            =  -0.01854 , ev
gamma4_19_19            =  -0.01122 , ev
gamma4_20_20            =  -0.05377 , ev
gamma4_21_21            =   0.05924 , ev
gamma4_22_22            =   0.05933 , ev
gamma4_23_23            =   0.05943 , ev
gamma5_1_1              =   0.01000 , ev
```

```
gamma5_2_2           =  -0.04084 , ev
gamma5_3_3           =  -0.02338 , ev
gamma5_4_4           =  -0.01014 , ev
gamma5_5_5           =  -0.03933 , ev
gamma5_6_6           =   0.03290 , ev
gamma5_7_7           =   0.00421 , ev
gamma5_8_8           =   0.01512 , ev
gamma5_9_9           =  -0.00175 , ev
gamma5_10_10         =  -0.02822 , ev
gamma5_11_11         =   0.00125 , ev
gamma5_12_12         =   0.00434 , ev
gamma5_13_13         =   0.08288 , ev
gamma5_14_14         =  -0.00739 , ev
gamma5_15_15         =  -0.00388 , ev
gamma5_16_16         =  -0.00091 , ev
gamma5_17_17         =  -0.00974 , ev
gamma5_18_18         =  -0.00707 , ev
gamma5_19_19         =  -0.04786 , ev
gamma5_20_20         =  -0.08293 , ev
gamma5_21_21         =   0.05903 , ev
gamma5_22_22         =   0.06151 , ev
gamma5_23_23         =   0.05831 , ev

gammax1_1_1           =   0.03194 , ev
gammax1_2_2           =  -0.02329 , ev
gammax1_3_3           =  -0.00909 , ev
gammax1_4_4           =   0.00585 , ev
```

```
gammax1_5_5              =  -0.02108 , ev
gammax1_6_6              =   0.03672 , ev
gammax1_7_7              =   0.00984 , ev
gammax1_8_8              =   0.01658 , ev
gammax1_9_9              =   0.00564 , ev
gammax1_10_10            =  -0.00098 , ev
gammax1_11_11            =   0.00226 , ev
gammax1_12_12            =  -0.00190 , ev
gammax1_13_13            =   0.06899 , ev
gammax1_14_14            =   0.00160 , ev
gammax1_15_15            =   0.00589 , ev
gammax1_16_16            =   0.00717 , ev
gammax1_17_17            =   0.00294 , ev
gammax1_18_18            =   0.00427 , ev
gammax1_19_19            =  -0.01735 , ev
gammax1_20_20            =   0.00373 , ev
gammax1_21_21            =   0.06303 , ev
gammax1_22_22            =   0.06235 , ev
gammax1_23_23            =   0.06198 , ev
gammax2_1_1              =   0.03257 , ev
gammax2_2_2              =  -0.02343 , ev
gammax2_3_3              =  -0.00902 , ev
gammax2_4_4              =   0.00565 , ev
gammax2_5_5              =  -0.02204 , ev
gammax2_6_6              =   0.03623 , ev
gammax2_7_7              =   0.00971 , ev
gammax2_8_8              =   0.01697 , ev
```

```
gammax2_9_9              =   0.00594 , ev
gammax2_10_10            =  -0.00098 , ev
gammax2_11_11            =   0.00195 , ev
gammax2_12_12            =  -0.00191 , ev
gammax2_13_13            =   0.06899 , ev
gammax2_14_14            =   0.00659 , ev
gammax2_15_15            =   0.00630 , ev
gammax2_16_16            =   0.00704 , ev
gammax2_17_17            =   0.00199 , ev
gammax2_18_18            =   0.00427 , ev
gammax2_19_19            =  -0.01742 , ev
gammax2_20_20            =   0.00361 , ev
gammax2_21_21            =   0.06252 , ev
gammax2_22_22            =   0.06479 , ev
gammax2_23_23            =   0.06137 , ev

# Diabatic curves with parameters

1D_24 =  18.843946229 , ev
1A_24 =   0.103785958
1X_24 =  -0.017811471
1E_24 =  -0.000064276 , ev
2D_24 =  16.605034747 , ev
2A_24 =   0.113697024
2X_24 =  -0.071525788
2E_24 =  -0.001089266 , ev
3D_24 =  24.183166560 , ev
```

```
3A_24 =   0.092830894

3X_24 =  -0.173402626

3E_24 =  -0.006166348 , ev

4D_24 =  18.704082166 , ev

4A_24 =   0.103455582

4X_24 =  -0.054194716

4E_24 =  -0.000584688 , ev

5D_24 =  19.274934324 , ev

5A_24 =   0.103723493

5X_24 =  -0.097783763

5E_24 =  -0.001962816 , ev


x1D_24 =  15.688146043 , ev

x1A_24 =   0.115075181

x1X_24 =  -0.180268278

x1E_24 =  -0.006612717 , ev

x2D_24 =  15.679824447 , ev

x2A_24 =   0.115111540

x2X_24 =  -0.182448296

x2E_24 =  -0.006772570 , ev


end-parameter-section


LABELS-SECTION


# Diabatic function labels
```

```
v1m24=morse1[1D_24,1A_24,1X_24,1E_24]
v2m24=morse1[2D_24,2A_24,2X_24,2E_24]
v3m24=morse1[3D_24,3A_24,3X_24,3E_24]
v4m24=morse1[4D_24,4A_24,4X_24,4E_24]
v5m24=morse1[5D_24,5A_24,5X_24,5E_24]

vx1m24=morse1[x1D_24,x1A_24,x1X_24,x1E_24]
vx2m24=morse1[x2D_24,x2A_24,x2X_24,x2E_24]

end-labels-section

HAMILTONIAN-SECTION
-------------------------------------------------------
modes| v1  | v2  | v3  | v4  | v5  | v6  | v7  | v8  | v9  | v10 |
modes| v11 | v12 | v13 | v14 | v15 | v16 | v17 | v18 | v19 | v20 |
modes| v21 | v22 | v23 | v24 | el
-------------------------------------------------------

# Kinetic Energy

omega_1               |1      KE
omega_2               |2      KE
omega_3               |3      KE
omega_4               |4      KE
omega_5               |5      KE
omega_6               |6      KE
omega_7               |7      KE
```

```
omega_8              |8     KE
omega_9              |9     KE
omega_10             |10    KE
omega_11             |11    KE
omega_12             |12    KE
omega_13             |13    KE
omega_14             |14    KE
omega_15             |15    KE
omega_16             |16    KE
omega_17             |17    KE
omega_18             |18    KE
omega_19             |19    KE
omega_20             |20    KE
omega_21             |21    KE
omega_22             |22    KE
omega_23             |23    KE
omega_24             |24    KE

# Potential for Harmonic oscillator


0.5*omega_1          |1     q^2
0.5*omega_2          |2     q^2
0.5*omega_3          |3     q^2
0.5*omega_4          |4     q^2
0.5*omega_5          |5     q^2
0.5*omega_6          |6     q^2
0.5*omega_7          |7     q^2
```

```
0.5*omega_8           |8     q^2
0.5*omega_9           |9     q^2
0.5*omega_10          |10    q^2
0.5*omega_11          |11    q^2
0.5*omega_12          |12    q^2
0.5*omega_13          |13    q^2
0.5*omega_14          |14    q^2
0.5*omega_15          |15    q^2
0.5*omega_16          |16    q^2
0.5*omega_17          |17    q^2
0.5*omega_18          |18    q^2
0.5*omega_19          |19    q^2
0.5*omega_20          |20    q^2
0.5*omega_21          |21    q^2
0.5*omega_22          |22    q^2
0.5*omega_23          |23    q^2

# Electronic States

E1                    |25    S1&1
E2                    |25    S2&2
E3                    |25    S3&3
E4                    |25    S4&4
E5                    |25    S5&5
E6                    |25    S6&6
E7                    |25    S7&7
E8                    |25    S8&8
```

```
E9                      |25     S9&9

E10                     |25     S10&10

E11                     |25     S11&11

E12                     |25     S12&12

E13                     |25     S13&13

E14                     |25     S14&14

E15                     |25     S15&15

E16                     |25     S16&16

E17                     |25     S17&17

E18                     |25     S18&18

E19                     |25     S19&19

E20                     |25     S20&20


X1                      |25     S21&21

X2                      |25     S22&22

-I*L_e                  |25     S21&21

-I*L_e                  |25     S22&22


# Lambda


lambda3_4_5             |5      q      |25     S3&4

lambda2_3_7             |7      q      |25     S2&3

lambda3_4_8             |8      q      |25     S3&4

lambda3_5_9             |9      q      |25     S3&5

lambda2_5_10            |10     q      |25     S2&5

lambda4_5_12            |12     q      |25     S4&5

lambda4_5_13            |13     q      |25     S4&5
```

```
lambda2_5_14        |14    q     |25    S2&5
lambda2_4_16        |16    q     |25    S2&4
lambda4_5_17        |17    q     |25    S4&5
lambda2_5_18        |18    q     |25    S2&5
lambda2_4_19        |19    q     |25    S2&4
lambda2_4_21        |21    q     |25    S2&4
lambda2_5_22        |22    q     |25    S2&5

lambda6_7_10        |10    q     |25    S6&7
lambda6_7_14        |14    q     |25    S6&7
lambda6_7_18        |18    q     |25    S6&7
lambda6_7_22        |22    q     |25    S6&7
lambda6_8_7         |7     q     |25    S6&8
lambda6_9_7         |7     q     |25    S6&9
lambda6_10_12       |12    q     |25    S6&10
lambda6_10_13       |13    q     |25    S6&10
lambda6_10_17       |17    q     |25    S6&10
lambda6_10_23       |23    q     |25    S6&10
lambda6_11_10       |10    q     |25    S6&11
lambda6_11_14       |14    q     |25    S6&11
lambda6_11_18       |18    q     |25    S6&11
lambda6_11_22       |22    q     |25    S6&11
lambda6_12_2        |2     q     |25    S6&12
lambda6_12_6        |6     q     |25    S6&12
lambda6_13_12       |12    q     |25    S6&13
lambda6_13_13       |13    q     |25    S6&13
lambda6_13_17       |17    q     |25    S6&13
```

lambda6_13_23	|23	q	|25	S6&13
lambda6_14_4	|4	q	|25	S6&14
lambda6_14_16	|16	q	|25	S6&14
lambda6_14_19	|19	q	|25	S6&14
lambda6_14_21	|21	q	|25	S6&14
lambda6_15_3	|3	q	|25	S6&15
lambda6_15_11	|11	q	|25	S6&15
lambda6_15_15	|15	q	|25	S6&15
lambda6_15_20	|20	q	|25	S6&15
lambda6_15_24	|24	q	|25	S6&15
lambda6_16_1	|1	q	|25	S6&16
lambda6_16_9	|9	q	|25	S6&16
lambda6_17_10	|10	q	|25	S6&17
lambda6_17_14	|14	q	|25	S6&17
lambda6_17_18	|18	q	|25	S6&17
lambda6_17_22	|22	q	|25	S6&17
lambda6_18_7	|7	q	|25	S6&18
lambda6_19_1	|1	q	|25	S6&19
lambda6_19_9	|9	q	|25	S6&19
lambda6_20_5	|5	q	|25	S6&20
lambda6_20_8	|8	q	|25	S6&20
lambda7_8_1	|1	q	|25	S7&8
lambda7_8_9	|9	q	|25	S7&8
lambda7_9_1	|1	q	|25	S7&9
lambda7_9_9	|9	q	|25	S7&9
lambda7_10_4	|4	q	|25	S7&10

```
lambda7_10_16      |16   q    |25   S7&10
lambda7_10_19      |19   q    |25   S7&10
lambda7_10_21      |21   q    |25   S7&10
lambda7_11_3       |3    q    |25   S7&11
lambda7_11_11      |11   q    |25   S7&11
lambda7_11_15      |15   q    |25   S7&11
lambda7_11_20      |20   q    |25   S7&11
lambda7_11_24      |24   q    |25   S7&11
lambda7_12_5       |5    q    |25   S7&12
lambda7_12_8       |8    q    |25   S7&12
lambda7_13_4       |4    q    |25   S7&13
lambda7_13_16      |16   q    |25   S7&13
lambda7_13_19      |19   q    |25   S7&13
lambda7_13_21      |21   q    |25   S7&13
lambda7_14_12      |12   q    |25   S7&14
lambda7_14_13      |13   q    |25   S7&14
lambda7_14_17      |17   q    |25   S7&14
lambda7_14_23      |23   q    |25   S7&14
lambda7_15_10      |10   q    |25   S7&15
lambda7_15_14      |14   q    |25   S7&15
lambda7_15_18      |18   q    |25   S7&15
lambda7_15_22      |22   q    |25   S7&15
lambda7_16_7       |7    q    |25   S7&16
lambda7_17_3       |3    q    |25   S7&17
lambda7_17_11      |11   q    |25   S7&17
lambda7_17_15      |15   q    |25   S7&17
lambda7_17_20      |20   q    |25   S7&17
```

lambda7_17_24	|24	q	|25	S7&17
lambda7_18_1	|1	q	|25	S7&18
lambda7_18_9	|9	q	|25	S7&18
lambda7_19_7	|7	q	|25	S7&19
lambda7_20_2	|2	q	|25	S7&20
lambda7_20_6	|6	q	|25	S7&20

lambda8_9_3	|3	q	|25	S8&9
lambda8_9_11	|11	q	|25	S8&9
lambda8_9_15	|15	q	|25	S8&9
lambda8_9_20	|20	q	|25	S8&9
lambda8_9_24	|24	q	|25	S8&9
lambda8_10_2	|2	q	|25	S8&10
lambda8_10_6	|6	q	|25	S8&10
lambda8_11_1	|1	q	|25	S8&11
lambda8_11_9	|9	q	|25	S8&11
lambda8_12_12	|12	q	|25	S8&12
lambda8_12_13	|13	q	|25	S8&12
lambda8_12_17	|17	q	|25	S8&12
lambda8_12_23	|23	q	|25	S8&12
lambda8_13_2	|2	q	|25	S8&13
lambda8_13_6	|6	q	|25	S8&13
lambda8_14_5	|5	q	|25	S8&14
lambda8_14_8	|8	q	|25	S8&14
lambda8_15_7	|7	q	|25	S8&15
lambda8_16_10	|10	q	|25	S8&16
lambda8_16_14	|14	q	|25	S8&16

lambda8_16_18	|18	q	|25	S8&16
lambda8_16_22	|22	q	|25	S8&16
lambda8_17_1	|1	q	|25	S8&17
lambda8_17_9	|9	q	|25	S8&17
lambda8_18_3	|3	q	|25	S8&18
lambda8_18_11	|11	q	|25	S8&18
lambda8_18_15	|15	q	|25	S8&18
lambda8_18_20	|20	q	|25	S8&18
lambda8_18_24	|24	q	|25	S8&18
lambda8_19_10	|10	q	|25	S8&19
lambda8_19_14	|14	q	|25	S8&19
lambda8_19_18	|18	q	|25	S8&19
lambda8_19_22	|22	q	|25	S8&19
lambda8_20_4	|4	q	|25	S8&20
lambda8_20_16	|16	q	|25	S8&20
lambda8_20_19	|19	q	|25	S8&20
lambda8_20_21	|21	q	|25	S8&20

lambda9_10_2	|2	q	|25	S9&10
lambda9_10_6	|6	q	|25	S9&10
lambda9_11_1	|1	q	|25	S9&11
lambda9_11_9	|9	q	|25	S9&11
lambda9_12_12	|12	q	|25	S9&12
lambda9_12_13	|13	q	|25	S9&12
lambda9_12_16	|16	q	|25	S9&12
lambda9_12_23	|23	q	|25	S9&12
lambda9_13_2	|2	q	|25	S9&13

```
lambda9_13_6      |6   q   |25   S9&13
lambda9_14_5      |5   q   |25   S9&14
lambda9_14_8      |8   q   |25   S9&14
lambda9_15_7      |7   q   |25   S9&15
lambda9_16_10     |10  q   |25   S9&16
lambda9_16_14     |14  q   |25   S9&16
lambda9_16_18     |18  q   |25   S9&16
lambda9_16_22     |22  q   |25   S9&16
lambda9_17_1      |1   q   |25   S9&17
lambda9_17_9      |9   q   |25   S9&17
lambda9_18_3      |3   q   |25   S9&18
lambda9_18_11     |11  q   |25   S9&18
lambda9_18_15     |15  q   |25   S9&18
lambda9_18_20     |20  q   |25   S9&18
lambda9_18_24     |24  q   |25   S9&18
lambda9_19_10     |10  q   |25   S9&19
lambda9_19_14     |14  q   |25   S9&19
lambda9_19_18     |18  q   |25   S9&19
lambda9_19_22     |22  q   |25   S9&19
lambda9_20_4      |4   q   |25   S9&20
lambda9_20_16     |16  q   |25   S9&20
lambda9_20_19     |19  q   |25   S9&20
lambda9_20_21     |21  q   |25   S9&20

lambda10_11_4     |4   q   |25   S10&11
lambda10_11_16    |16  q   |25   S10&11
lambda10_11_19    |19  q   |25   S10&11
```

```
lambda10_11_21      |21     q      |25     S10&11
lambda10_12_7       |7      q      |25     S10&12
lambda10_13_3       |3      q      |25     S10&13
lambda10_13_11      |11     q      |25     S10&13
lambda10_13_15      |15     q      |25     S10&13
lambda10_13_20      |20     q      |25     S10&13
lambda10_13_24      |24     q      |25     S10&13
lambda10_14_10      |10     q      |25     S10&14
lambda10_14_14      |14     q      |25     S10&14
lambda10_14_18      |18     q      |25     S10&14
lambda10_14_22      |22     q      |25     S10&14
lambda10_15_12      |12     q      |25     S10&15
lambda10_15_13      |13     q      |25     S10&15
lambda10_15_17      |17     q      |25     S10&15
lambda10_15_23      |23     q      |25     S10&15
lambda10_16_5       |5      q      |25     S10&16
lambda10_16_8       |8      q      |25     S10&16
lambda10_17_4       |4      q      |25     S10&17
lambda10_17_16      |16     q      |25     S10&17
lambda10_17_17      |17     q      |25     S10&17
lambda10_17_21      |21     q      |25     S10&17
lambda10_18_2       |2      q      |25     S10&18
lambda10_18_6       |6      q      |25     S10&18
lambda10_19_5       |5      q      |25     S10&19
lambda10_19_8       |8      q      |25     S10&19
lambda10_20_1       |1      q      |25     S10&20
lambda10_20_9       |9      q      |25     S10&20
```

```
lambda11_12_5     |5    q   |25   S11&12
lambda11_12_8     |8    q   |25   S11&12
lambda11_13_4     |4    q   |25   S11&13
lambda11_13_16    |16   q   |25   S11&13
lambda11_13_19    |19   q   |25   S11&13
lambda11_13_21    |21   q   |25   S11&13
lambda11_14_12    |12   q   |25   S11&14
lambda11_14_13    |13   q   |25   S11&14
lambda11_14_17    |17   q   |25   S11&14
lambda11_14_23    |23   q   |25   S11&14
lambda11_15_10    |10   q   |25   S11&15
lambda11_15_14    |14   q   |25   S11&15
lambda11_15_18    |18   q   |25   S11&15
lambda11_15_22    |22   q   |25   S11&15
lambda11_16_7     |7    q   |25   S11&16
lambda11_17_3     |3    q   |25   S11&17
lambda11_17_11    |11   q   |25   S11&17
lambda11_17_15    |15   q   |25   S11&17
lambda11_17_20    |20   q   |25   S11&17
lambda11_17_24    |24   q   |25   S11&17
lambda11_18_1     |1    q   |25   S11&18
lambda11_18_9     |9    q   |25   S11&18
lambda11_19_7     |7    q   |25   S11&19
lambda11_20_2     |2    q   |25   S11&20
lambda11_20_6     |6    q   |25   S11&20
```

```
lambda12_13_7        |7     q     |25     S12&13

lambda12_14_1        |1     q     |25     S12&14

lambda12_14_9        |9     q     |25     S12&14

lambda12_15_2        |2     q     |25     S12&15

lambda12_15_6        |6     q     |25     S12&15

lambda12_16_4        |4     q     |25     S12&16

lambda12_16_16       |16    q     |25     S12&16

lambda12_16_19       |19    q     |25     S12&16

lambda12_16_21       |21    q     |25     S12&16

lambda12_17_5        |5     q     |25     S12&17

lambda12_17_8        |8     q     |25     S12&17

lambda12_18_12       |12    q     |25     S12&18

lambda12_18_13       |13    q     |25     S12&18

lambda12_18_17       |17    q     |25     S12&18

lambda12_18_23       |23    q     |25     S12&18

lambda12_19_4        |4     q     |25     S12&19

lambda12_19_16       |16    q     |25     S12&19

lambda12_19_19       |19    q     |25     S12&19

lambda12_19_21       |21    q     |25     S12&19

lambda12_20_10       |10    q     |25     S12&20

lambda12_20_14       |14    q     |25     S12&20

lambda12_20_18       |18    q     |25     S12&20

lambda12_20_22       |22    q     |25     S12&20


lambda13_14_10       |10    q     |25     S13&14

lambda13_14_14       |14    q     |25     S13&14

lambda13_14_18       |18    q     |25     S13&14
```

```
lambda13_14_22     |22    q    |25    S13&14
lambda13_15_12     |12    q    |25    S13&15
lambda13_15_13     |13    q    |25    S13&15
lambda13_15_17     |17    q    |25    S13&15
lambda13_15_23     |23    q    |25    S13&15
lambda13_16_5      |5     q    |25    S13&16
lambda13_16_8      |8     q    |25    S13&16
lambda13_17_4      |4     q    |25    S13&17
lambda13_17_16     |16    q    |25    S13&17
lambda13_17_19     |19    q    |25    S13&17
lambda13_17_21     |21    q    |25    S13&17
lambda13_18_2      |2     q    |25    S13&18
lambda13_18_6      |6     q    |25    S13&18
lambda13_19_5      |5     q    |25    S13&19
lambda13_19_8      |8     q    |25    S13&19
lambda13_20_1      |1     q    |25    S13&20
lambda13_20_9      |9     q    |25    S13&20

lambda14_15_4      |4     q    |25    S14&15
lambda14_15_16     |16    q    |25    S14&15
lambda14_15_19     |19    q    |25    S14&15
lambda14_15_21     |21    q    |25    S14&15
lambda14_16_2      |2     q    |25    S14&16
lambda14_16_6      |6     q    |25    S14&16
lambda14_17_12     |12    q    |25    S14&17
lambda14_17_13     |13    q    |25    S14&17
lambda14_17_17     |17    q    |25    S14&17
```

```
lambda14_17_23      |23     q      |25     S14&17

lambda14_18_5       |5      q      |25     S14&18

lambda14_18_8       |8      q      |25     S14&18

lambda14_19_2       |2      q      |25     S14&19

lambda14_19_6       |6      q      |25     S14&19

lambda14_20_7       |7      q      |25     S14&20


lambda15_16_1       |1      q      |25     S15&16

lambda15_16_9       |9      q      |25     S15&16

lambda15_17_10      |10     q      |25     S15&17

lambda15_17_14      |14     q      |25     S15&17

lambda15_17_18      |18     q      |25     S15&17

lambda15_17_22      |22     q      |25     S15&17

lambda15_18_7       |7      q      |25     S15&18

lambda15_19_1       |1      q      |25     S15&19

lambda15_19_9       |9      q      |25     S15&19

lambda15_20_5       |5      q      |25     S15&20

lambda15_20_8       |8      q      |25     S15&20


lambda16_17_7       |7      q      |25     S16&17

lambda16_18_10      |10     q      |25     S16&18

lambda16_18_14      |14     q      |25     S16&18

lambda16_18_18      |18     q      |25     S16&18

lambda16_18_22      |22     q      |25     S16&18

lambda16_19_3       |3      q      |25     S16&19

lambda16_19_11      |11     q      |25     S16&19

lambda16_19_15      |15     q      |25     S16&19
```

```
lambda16_19_20        |20     q      |25     S16&19
lambda16_19_24        |24     q      |25     S16&19
lambda16_20_12        |12     q      |25     S16&20
lambda16_20_13        |13     q      |25     S16&20
lambda16_20_17        |17     q      |25     S16&20
lambda16_20_23        |23     q      |25     S16&20

lambda17_18_1         |1      q      |25     S17&18
lambda17_18_9         |9      q      |25     S17&18
lambda17_19_7         |7      q      |25     S17&19
lambda17_20_2         |2      q      |25     S17&20
lambda17_20_6         |6      q      |25     S17&20

lambda18_19_10        |10     q      |25     S18&19
lambda18_19_14        |14     q      |25     S18&19
lambda18_19_17        |17     q      |25     S18&19
lambda18_19_22        |22     q      |25     S18&19
lambda18_20_4         |4      q      |25     S18&20
lambda18_20_16        |16     q      |25     S18&20
lambda18_20_19        |19     q      |25     S18&20
lambda18_20_21        |21     q      |25     S18&20

lambda19_20_12        |12     q      |25     S19&20
lambda19_20_13        |13     q      |25     S19&20
lambda19_20_17        |17     q      |25     S19&20
lambda19_20_23        |23     q      |25     S19&20
```

```
lambdax1_x2_10        |10    q     |25    S21&22
lambdax1_x2_14        |14    q     |25    S21&22
lambdax1_x2_18        |18    q     |25    S21&22
lambdax1_x2_22        |22    q     |25    S21&22

# Kappa

kappa2_3              |3     q     |25    S2&2
kappa3_3              |3     q     |25    S3&3
kappa4_3              |3     q     |25    S4&4
kappa5_3              |3     q     |25    S5&5
kappa2_11             |11    q     |25    S2&2
kappa3_11             |11    q     |25    S3&3
kappa4_11             |11    q     |25    S4&4
kappa5_11             |11    q     |25    S5&5
kappa2_15             |15    q     |25    S2&2
kappa3_15             |15    q     |25    S3&3
kappa4_15             |15    q     |25    S4&4
kappa5_15             |15    q     |25    S5&5
kappa2_20             |20    q     |25    S2&2
kappa3_20             |20    q     |25    S3&3
kappa4_20             |20    q     |25    S4&4
kappa5_20             |20    q     |25    S5&5

kappa6_3              |3     q     |25    S6&6
kappa6_11             |11    q     |25    S6&6
kappa6_15             |15    q     |25    S6&6
```

```
kappa6_20       |20    q    |25    S6&6
kappa6_24       |24    q    |25    S6&6
kappa7_3        |3     q    |25    S7&7
kappa7_11       |11    q    |25    S7&7
kappa7_15       |15    q    |25    S7&7
kappa7_20       |20    q    |25    S7&7
kappa7_24       |24    q    |25    S7&7
kappa8_3        |3     q    |25    S8&8
kappa8_11       |11    q    |25    S8&8
kappa8_15       |15    q    |25    S8&8
kappa8_20       |20    q    |25    S8&8
kappa8_24       |24    q    |25    S8&8
kappa9_3        |3     q    |25    S9&9
kappa9_11       |11    q    |25    S9&9
kappa9_15       |15    q    |25    S9&9
kappa9_20       |20    q    |25    S9&9
kappa9_24       |24    q    |25    S9&9
kappa10_3       |3     q    |25    S10&10
kappa10_11      |11    q    |25    S10&10
kappa10_15      |15    q    |25    S10&10
kappa10_20      |20    q    |25    S10&10
kappa10_24      |24    q    |25    S10&10
kappa11_3       |3     q    |25    S11&11
kappa11_11      |11    q    |25    S11&11
kappa11_15      |15    q    |25    S11&11
kappa11_20      |20    q    |25    S11&11
kappa11_24      |24    q    |25    S11&11
```

```
kappa12_3      |3    q    |25    S12&12
kappa12_11     |11   q    |25    S12&12
kappa12_15     |15   q    |25    S12&12
kappa12_20     |20   q    |25    S12&12
kappa12_24     |24   q    |25    S12&12
kappa13_3      |3    q    |25    S13&13
kappa13_11     |11   q    |25    S13&13
kappa13_15     |15   q    |25    S13&13
kappa13_20     |20   q    |25    S13&13
kappa13_24     |24   q    |25    S13&13
kappa14_3      |3    q    |25    S14&14
kappa14_11     |11   q    |25    S14&14
kappa14_15     |15   q    |25    S14&14
kappa14_20     |20   q    |25    S14&14
kappa14_24     |24   q    |25    S14&14
kappa15_3      |3    q    |25    S15&15
kappa15_11     |11   q    |25    S15&15
kappa15_15     |15   q    |25    S15&15
kappa15_20     |20   q    |25    S15&15
kappa15_24     |24   q    |25    S15&15
kappa16_3      |3    q    |25    S16&16
kappa16_11     |11   q    |25    S16&16
kappa16_15     |15   q    |25    S16&16
kappa16_20     |20   q    |25    S16&16
kappa16_24     |24   q    |25    S16&16
kappa17_3      |3    q    |25    S17&17
kappa17_11     |11   q    |25    S17&17
```

```
kappa17_15          |15     q     |25     S17&17
kappa17_20          |20     q     |25     S17&17
kappa17_24          |24     q     |25     S17&17
kappa18_3           |3      q     |25     S18&18
kappa18_11          |11     q     |25     S18&18
kappa18_15          |15     q     |25     S18&18
kappa18_20          |20     q     |25     S18&18
kappa18_24          |24     q     |25     S18&18
kappa19_3           |3      q     |25     S19&19
kappa19_11          |11     q     |25     S19&19
kappa19_15          |15     q     |25     S19&19
kappa19_20          |20     q     |25     S19&19
kappa19_24          |24     q     |25     S19&19
kappa20_3           |3      q     |25     S20&20
kappa20_11          |11     q     |25     S20&20
kappa20_15          |15     q     |25     S20&20
kappa20_20          |20     q     |25     S20&20
kappa20_24          |24     q     |25     S20&20

kappax1_3           |3      q     |25     S21&21
kappax1_11          |11     q     |25     S21&21
kappax1_15          |15     q     |25     S21&21
kappax1_20          |20     q     |25     S21&21
kappax2_3           |3      q     |25     S22&22
kappax2_11          |11     q     |25     S22&22
kappax2_15          |15     q     |25     S22&22
kappax2_20          |20     q     |25     S22&22
```

```
# On Diagonal Gamma Constants

0.5*gamma2_1_1        |1     q^2     |25     S2&2
0.5*gamma3_1_1        |1     q^2     |25     S3&3
0.5*gamma4_1_1        |1     q^2     |25     S4&4
0.5*gamma5_1_1        |1     q^2     |25     S5&5
0.5*gamma2_2_2        |2     q^2     |25     S2&2
0.5*gamma3_2_2        |2     q^2     |25     S3&3
0.5*gamma4_2_2        |2     q^2     |25     S4&4
0.5*gamma5_2_2        |2     q^2     |25     S5&5
0.5*gamma2_3_3        |3     q^2     |25     S2&2
0.5*gamma3_3_3        |3     q^2     |25     S3&3
0.5*gamma4_3_3        |3     q^2     |25     S4&4
0.5*gamma5_3_3        |3     q^2     |25     S5&5
0.5*gamma2_4_4        |4     q^2     |25     S2&2
0.5*gamma3_4_4        |4     q^2     |25     S3&3
0.5*gamma4_4_4        |4     q^2     |25     S4&4
0.5*gamma5_4_4        |4     q^2     |25     S5&5
0.5*gamma2_5_5        |5     q^2     |25     S2&2
0.5*gamma3_5_5        |5     q^2     |25     S3&3
0.5*gamma4_5_5        |5     q^2     |25     S4&4
0.5*gamma5_5_5        |5     q^2     |25     S5&5
0.5*gamma2_6_6        |6     q^2     |25     S2&2
0.5*gamma3_6_6        |6     q^2     |25     S3&3
0.5*gamma4_6_6        |6     q^2     |25     S4&4
0.5*gamma5_6_6        |6     q^2     |25     S5&5
```

```
0.5*gamma2_7_7        |7     q^2      |25     S2&2
0.5*gamma3_7_7        |7     q^2      |25     S3&3
0.5*gamma4_7_7        |7     q^2      |25     S4&4
0.5*gamma5_7_7        |7     q^2      |25     S5&5
0.5*gamma2_8_8        |8     q^2      |25     S2&2
0.5*gamma3_8_8        |8     q^2      |25     S3&3
0.5*gamma4_8_8        |8     q^2      |25     S4&4
0.5*gamma5_8_8        |8     q^2      |25     S5&5
0.5*gamma2_9_9        |9     q^2      |25     S2&2
0.5*gamma3_9_9        |9     q^2      |25     S3&3
0.5*gamma4_9_9        |9     q^2      |25     S4&4
0.5*gamma5_9_9        |9     q^2      |25     S5&5
0.5*gamma2_10_10      |10    q^2      |25     S2&2
0.5*gamma3_10_10      |10    q^2      |25     S3&3
0.5*gamma4_10_10      |10    q^2      |25     S4&4
0.5*gamma5_10_10      |10    q^2      |25     S5&5
0.5*gamma2_11_11      |11    q^2      |25     S2&2
0.5*gamma3_11_11      |11    q^2      |25     S3&3
0.5*gamma4_11_11      |11    q^2      |25     S4&4
0.5*gamma5_11_11      |11    q^2      |25     S5&5
0.5*gamma2_12_12      |12    q^2      |25     S2&2
0.5*gamma3_12_12      |12    q^2      |25     S3&3
0.5*gamma4_12_12      |12    q^2      |25     S4&4
0.5*gamma5_12_12      |12    q^2      |25     S5&5
0.5*gamma2_13_13      |13    q^2      |25     S2&2
0.5*gamma3_13_13      |13    q^2      |25     S3&3
0.5*gamma4_13_13      |13    q^2      |25     S4&4
```

```
0.5*gamma5_13_13       |13     q^2       |25     S5&5
0.5*gamma2_14_14       |14     q^2       |25     S2&2
0.5*gamma3_14_14       |14     q^2       |25     S3&3
0.5*gamma4_14_14       |14     q^2       |25     S4&4
0.5*gamma5_14_14       |14     q^2       |25     S5&5
0.5*gamma2_15_15       |15     q^2       |25     S2&2
0.5*gamma3_15_15       |15     q^2       |25     S3&3
0.5*gamma4_15_15       |15     q^2       |25     S4&4
0.5*gamma5_15_15       |15     q^2       |25     S5&5
0.5*gamma2_16_16       |16     q^2       |25     S2&2
0.5*gamma3_16_16       |16     q^2       |25     S3&3
0.5*gamma4_16_16       |16     q^2       |25     S4&4
0.5*gamma5_16_16       |16     q^2       |25     S5&5
0.5*gamma2_17_17       |17     q^2       |25     S2&2
0.5*gamma3_17_17       |17     q^2       |25     S3&3
0.5*gamma4_17_17       |17     q^2       |25     S4&4
0.5*gamma5_17_17       |17     q^2       |25     S5&5
0.5*gamma2_18_18       |18     q^2       |25     S2&2
0.5*gamma3_18_18       |18     q^2       |25     S3&3
0.5*gamma4_18_18       |18     q^2       |25     S4&4
0.5*gamma5_18_18       |18     q^2       |25     S5&5
0.5*gamma2_19_19       |19     q^2       |25     S2&2
0.5*gamma3_19_19       |19     q^2       |25     S3&3
0.5*gamma4_19_19       |19     q^2       |25     S4&4
0.5*gamma5_19_19       |19     q^2       |25     S5&5
0.5*gamma2_20_20       |20     q^2       |25     S2&2
0.5*gamma3_20_20       |20     q^2       |25     S3&3
```

```
0.5*gamma4_20_20     |20    q^2     |25    S4&4

0.5*gamma5_20_20     |20    q^2     |25    S5&5

0.5*gamma2_21_21     |21    q^2     |25    S2&2

0.5*gamma3_21_21     |21    q^2     |25    S3&3

0.5*gamma4_21_21     |21    q^2     |25    S4&4

0.5*gamma5_21_21     |21    q^2     |25    S5&5

0.5*gamma2_22_22     |22    q^2     |25    S2&2

0.5*gamma3_22_22     |22    q^2     |25    S3&3

0.5*gamma4_22_22     |22    q^2     |25    S4&4

0.5*gamma5_22_22     |22    q^2     |25    S5&5

0.5*gamma2_23_23     |23    q^2     |25    S2&2

0.5*gamma3_23_23     |23    q^2     |25    S3&3

0.5*gamma4_23_23     |23    q^2     |25    S4&4

0.5*gamma5_23_23     |23    q^2     |25    S5&5


0.5*gammax1_1_1      |1     q^2     |25    S21&21

0.5*gammax1_2_2      |2     q^2     |25    S21&21

0.5*gammax1_3_3      |3     q^2     |25    S21&21

0.5*gammax1_4_4      |4     q^2     |25    S21&21

0.5*gammax1_5_5      |5     q^2     |25    S21&21

0.5*gammax1_6_6      |6     q^2     |25    S21&21

0.5*gammax1_7_7      |7     q^2     |25    S21&21

0.5*gammax1_8_8      |8     q^2     |25    S21&21

0.5*gammax1_9_9      |9     q^2     |25    S21&21

0.5*gammax1_10_10    |10    q^2     |25    S21&21

0.5*gammax1_11_11    |11    q^2     |25    S21&21

0.5*gammax1_12_12    |12    q^2     |25    S21&21
```

```
0.5*gammax1_13_13      |13    q^2     |25    S21&21
0.5*gammax1_14_14      |14    q^2     |25    S21&21
0.5*gammax1_15_15      |15    q^2     |25    S21&21
0.5*gammax1_16_16      |16    q^2     |25    S21&21
0.5*gammax1_17_17      |17    q^2     |25    S21&21
0.5*gammax1_18_18      |18    q^2     |25    S21&21
0.5*gammax1_19_19      |19    q^2     |25    S21&21
0.5*gammax1_20_20      |20    q^2     |25    S21&21
0.5*gammax1_21_21      |21    q^2     |25    S21&21
0.5*gammax1_22_22      |22    q^2     |25    S21&21
0.5*gammax1_23_23      |23    q^2     |25    S21&21
0.5*gammax2_1_1        |1     q^2     |25    S22&22
0.5*gammax2_2_2        |2     q^2     |25    S22&22
0.5*gammax2_3_3        |3     q^2     |25    S22&22
0.5*gammax2_4_4        |4     q^2     |25    S22&22
0.5*gammax2_5_5        |5     q^2     |25    S22&22
0.5*gammax2_6_6        |6     q^2     |25    S22&22
0.5*gammax2_7_7        |7     q^2     |25    S22&22
0.5*gammax2_8_8        |8     q^2     |25    S22&22
0.5*gammax2_9_9        |9     q^2     |25    S22&22
0.5*gammax2_10_10      |10    q^2     |25    S22&22
0.5*gammax2_11_11      |11    q^2     |25    S22&22
0.5*gammax2_12_12      |12    q^2     |25    S22&22
0.5*gammax2_13_13      |13    q^2     |25    S22&22
0.5*gammax2_14_14      |14    q^2     |25    S22&22
0.5*gammax2_15_15      |15    q^2     |25    S22&22
0.5*gammax2_16_16      |16    q^2     |25    S22&22
```

```
0.5*gammax2_17_17     |17    q^2     |25    S22&22

0.5*gammax2_18_18     |18    q^2     |25    S22&22

0.5*gammax2_19_19     |19    q^2     |25    S22&22

0.5*gammax2_20_20     |20    q^2     |25    S22&22

0.5*gammax2_21_21     |21    q^2     |25    S22&22

0.5*gammax2_22_22     |22    q^2     |25    S22&22

0.5*gammax2_23_23     |23    q^2     |25    S22&22


# Morse/Anti-Morse potential


 1.0   |24     v1m24     |25     S1&1

 1.0   |24     v2m24     |25     S2&2

 1.0   |24     v3m24     |25     S3&3

 1.0   |24     v4m24     |25     S4&4

 1.0   |24     v5m24     |25     S5&5


 1.0   |24     vx1m24     |25     S21&21

 1.0   |24     vx2m24     |25     S22&22


END-HAMILTONIAN-SECTION


END-OPERATOR
```
